# Ginsenoside Rg3 Reduces the Toxicity of Graphene Oxide Used for pH-Responsive Delivery of Doxorubicin to Liver and Breast Cancer Cells

**DOI:** 10.3390/pharmaceutics15020391

**Published:** 2023-01-24

**Authors:** Shadi Rahimi, Daniel van Leeuwen, Fariba Roshanzamir, Santosh Pandit, Lei Shi, Nima Sasanian, Jens Nielsen, Elin K. Esbjörner, Ivan Mijakovic

**Affiliations:** 1 Division of Systems and Synthetic Biology, Department of Biology and Biological Engineering, Chalmers University of Technology, 412 96 Göteborg, Sweden; 2 Division of Chemical Biology, Department of Biology and Biological Engineering, Chalmers University of Technology, 412 96 Göteborg, Sweden; 3 BioInnovation Institute, DK-2200 Copenhagen, Denmark; 4 The Novo Nordisk Foundation Center for Biosustainability, Technical University of Denmark, DK-2800 Lyngby, Denmark

**Keywords:** doxorubicin, drug carrier, drug delivery, ginsenoside Rg3, graphene oxide

## Abstract

Doxorubicin (DOX) is extensively used in chemotherapy, but it has serious side effects and is inefficient against some cancers, e.g., hepatocarcinoma. To ameliorate the delivery of DOX and reduce its side effects, we designed a pH-responsive delivery system based on graphene oxide (GO) that is capable of a targeted drug release in the acidic tumor microenvironment. GO itself disrupted glutathione biosynthesis and induced reactive oxygen species (ROS) accumulation in human cells. It induced IL17-directed JAK-STAT signaling and VEGF gene expression, leading to increased cell proliferation as an unwanted effect. To counter this, GO was conjugated with the antioxidant, ginsenoside Rg3, prior to loading with DOX. The conjugation of Rg3 to GO significantly reduced the toxicity of the GO carrier by abolishing ROS production. Furthermore, treatment of cells with GO–Rg3 did not induce IL17-directed JAK-STAT signaling and VEGF gene expression—nor cell proliferation—suggesting GO–Rg3 as a promising drug carrier. The anticancer activity of GO–Rg3–DOX conjugates was investigated against Huh7 hepatocarcinoma and MDA-MB-231 breast cancer cells. GO–Rg3–DOX conjugates significantly reduced cancer cell viability, primarily via downregulation of transcription regulatory genes and upregulation of apoptosis genes. GO–Rg3 is an effective, biocompatible, and pH responsive DOX carrier with potential to improve chemotherapy—at least against liver and breast cancers.

## 1. Introduction

Cancer is a major global healthcare problem with 9.6 million deaths reported in 2018 [1]. Various chemotherapy techniques, including doxorubicin (DOX) chemotherapy, are clinically applied to promote patient survival by suppressing tumor proliferation [2]. One of the caveats of DOX chemotherapy is its lack of selectivity, which results in deleterious side effects throughout the patient’s body [3]. The therapeutic utilization of DOX is limited due to its cardiotoxicity and nephrotoxicity [4,5]. DOX-induced cellular injury involves lipid peroxidation, changes in adenylate cyclase activity, and the formation of reactive oxygen species (ROS), thereby leading to DOX-induced inflammation and apoptosis [4]. To improve DOX chemotherapy’s efficacy and selectivity, various innovative drug delivery systems have been evaluated over the past few decades [6]. However, the issues with DOX’s lack of selectivity and side effects are still not resolved.

One family of efficient drug delivery systems comprises graphene-based water-soluble nanosheets. Among these, graphene oxide (GO) sheets have received considerable attention [7,8]. GO, an oxidized form of graphene, shows good biocompatibility and water dispersibility [9]. The hexagonal rings of carbon atoms in GO bear oxygen functional groups, such as carboxylic, hydroxyl, and epoxide groups, that are primarily distributed on the edges of the sheet. In addition to improving water dispersibility, these functional groups enhance the capacity of GO to reversibly interact with different types of drugs. This property can be used to achieve controlled drug delivery and release [10]. There are several studies on using GO as the platform for the delivery of DOX [11,12,13,14,15]. GO is an advantageous DOX carrier due to its pH-dependent interaction with the drug, favoring DOX release in the acidic tumor microenvironment. Specifically, the protonation of amino groups on DOX leads to dissociation from GO, and the hydrogen-bonding interactions between GO and DOX are also weakened at an acidic pH [16]. However, GO itself induces the unwanted generation of intracellular ROS in a concentration and time-dependent manner [8,17]. 

However, the versatility of the GO surface allows for the integration of several types of molecules into a single drug delivery nanoplatform [18,19], and this possibility is explored in our study. Ginsenoside Rg3, a tetracyclic triterpenoid saponin, is an active ingredient present in *Panax ginseng* C.A. Meyer (Korean ginseng plant). It is well known for its effects in promoting homeostasis [20] and it has been explored as a potential anticancer drug. Rg3 was found to inhibit tumor growth and metastasis in mice and tumor cell invasion in vitro, as well as enhancing the body’s immunity [21,22,23,24,25,26,27,28]. The anti-tumor drug, Rg3 Shenyi Capsule, which mainly consists of ginsenoside Rg3, was approved by the State Food and Drug Administration in China in 2003, and it is used to inhibit and prevent cancers [29]. There are several clinical studies showing the efficacy of treatment with ginsenoside Rg3 combined with classical chemotherapy [30,31,32]. Besides its own anticancer effects, Rg3 has been found to mitigate DOX-induced cardiotoxicity [33,34]. Hence, we identified it as an interesting additive to our DOX carrier nanoplatform, primarily to mitigate GO-induced oxidative stress. 

In this study, we investigated a combined delivery of Rg3 and DOX into human cancer cells using a GO-based nanoplatform. The GO–Rg3–DOX nanoplatform was effective against human Huh7 hepatocarcinoma and MDA-MB-231 breast cancer cells. Rg3 specifically contributes to the reduction of unwanted ROS accumulation induced by the GO carrier, thus mitigating unwanted side effects. Optimal DOX release from GO–Rg3–DOX was achieved in acidic conditions, leading to a targeted release in the acidic tumor microenvironment. Our RNA sequencing experiments revealed that the killing effect of the GO–Rg3–DOX nanoplatform proceeds primarily by downregulation of transcription regulatory genes, extracellular matrix degradation, and apoptosis. This confirmed that a specific effect against targeted cancer cells was achieved, while the ROS stress, which leads to systemic side effects, was minimized by the presence of Rg3.

## 2. Materials and Methods

### 2.1. Cell Culture

All experiments were performed with mycoplasma-free cells. All human cell lines have been authenticated using STR (or SNP) profiling within the last three years. Human MDA-MB-231 breast cancer cells were purchased from ATCC. Human Huh7 hepatoma cells were a kind gift from Prof. Samir El-Andaloussi, Karolinska Institute. All cell lines were maintained at 37 °C with 5% CO_2_ in Dulbecco’s Modified Eagle’s Medium (Thermo Scientific, Waltham, MA, USA) containing 4500 g/mL glucose, which was further supplemented with 10% fetal bovine serum (Thermo Scientific), 100 U/mL of penicillin, and 100 μg/mL of streptomycin (Sigma Aldrich, St. Louis, MO, USA).

### 2.2. Preparation of GO–Rg3 and GO–Rg3–DOX Conjugates 

For preparation of GO–Rg3, 2 mg/mL (1 mM) of ultra-highly concentrated single-layer GO (Graphene Supermarket) and 0.5 or 1 mg/mL (0.6–1.2 mM) of ginsenoside Rg3 (Sigma) were mixed, and 10% concentrated sulfuric acid (Merck) was carefully poured down the walls of the flask. Esterification is a relatively slow process at room temperature and did not proceed to completion. Concentrated sulfuric acid was used as a catalyst and had a dual role: speeding up the reaction and acting as a dehydrating agent, thereby forcing the equilibrium to the right and resulting in a greater yield of ester.

Afterwards, the reactants were mixed and the mixture was heated at reflux with a thermowell for 3 h at 80 °C. Then, the reaction mixture was cooled to room temperature and subsequently poured into water and centrifuged. After centrifuging several times with water, the supernatant’s pH reached neutral [35]. For subsequent DOX (Sigma) loading, GO–Rg3 solution in water (~2 mg/mL or 1 mM) was mixed with 0.1 mg/mL (0.2 mM) of DOX solution in water, and then the volume was adjusted to 50 mL at pH 8 overnight. Thus, the final DOX concentration in solution was kept low, around 0.004 mM, to avoid DOX self-aggregation [36,37]. Finally, the reaction mixture was washed several times with water. 

### 2.3. Characterization of GO, GO–Rg3, and GO–Rg3–DOX Conjugates

Infrared spectra of the samples were recorded using an attenuated total reflection Alpha Fourier-transform infrared spectroscopy (FTIR) from Bruker, with a diamond crystal as the refractive element, in the range of 350−4000 cm^−1^ at a resolution of 4 cm^−1^. 

Raman spectra were measured with a Raman microscope-WITec alpha300R (Ulm, Germany) equipped with a 50× objective and a 532 nm laser and 600 g/mm grating. Each spectrum was recorded in the range of 500–3000 cm^−1^ with 10 number accumulations and 0.5 s integration time. 

The freshly cleaved mica surface was incubated with 10 µL of 0.5% (*v*/*v*) (3-aminopropyl) triethoxysilane (APTES, Sigma Aldrich) for 1 min. Then, the mica surface was rinsed 5 times with 1 mL of Mili-Q water and dried under a gentle stream of nitrogen gas. Atomic force microscopy (AFM) samples at 10 µg mL^−1^ were prepared by depositing 10 µL of solution onto freshly cleaved mica, followed by a 15 min incubation to allow GO, GO–Rg3, and GO–Rg3–DOX to settle on the surface. The mica surfaces were thereafter rinsed 5 times with 1 mL of Milli-Q water and dried under a gentle stream of nitrogen gas. Images were obtained using an NTEGRA Prima (NT-MDT, Moscow, Russia) set-up equipped with a gold-coated single crystal silicon cantilever (NT-MDT, NSG01) with a resonance frequency of 150 kHz in tapping-mode. Images were recorded in 512 × 512 pixels, at a 0.5 Hz scan rate, and then processed using the Gwyddion software package 2.58: polynomial background subtraction followed by planar subtraction and adjustment of linear aberrations. Individual length was measured manually while the height profile was measured perpendicularly to the axis, and the associated mica background was subtracted.

The GO, GO–Rg3, and GO–Rg3–DOX size was measured by dynamic light scattering (DLS) on a Zetasizer nano (Malvern Panalytical Ltd., Malvern, UK) at room temperature using plastic cuvettes at a concentration of 10 µg mL^−1^. The water used as the dispersant and dispersant refractive index and viscosity were 1.330 and 0.8872 centipoise. The zeta potential of GO, GO–Rg3, and GO–Rg3–DOX were also measured on the Zetasizer at room temperature at a concentration of 10 µg mL^−1^.

### 2.4. Viability Assay Using Alamarblue Assay

Both MDA-MB-231 breast cancer cells and Huh7 human hepatoma cell lines were used for this study. Cells were seeded onto 96-well plates at a density of 2 × 10^4^ cells per well and cultured for 24 h before treatment with GO, GO–Rg3, GO–Rg3–DOX, and DOX for 24 h. Then, the cells were incubated with medium containing 1× alamarBlue (Thermo Scientific) staining solution for 6 h. The signal from the cells was detected using an OPTIMA BLUE Fluostar plate reader (BMG labtech, Ortenberg, Germany) and the results were normalized to the medium control. The positive control was 10% DMSO. 

### 2.5. Drug Release Analysis 

The SnakeSkin™ Dialysis Tubing (10 K MWCO, 16 mm) (Thermo Scientific) was filled with 1 mL of 100 µg/mL GO–Rg3–DOX suspended in 20 mL of PBS (Thermo Scientific) buffer at pH 5.5 and pH 7.4. A total of 5 mL of PBS buffer was collected at different time points (1, 2, 4, 6, and 12 days) and 5 mL of fresh PBS was replaced. The collected samples were first freeze-dried and then resuspended in water to eliminate the effect of pH on subsequent measurement. 

The HPLC system consisted of a Dionex UHPLC-PDA-FLD and a column AAA C-18 5 mm (150 mm × 4.6 mm) (AB Sciex Pte. Ltd., Framingham, MA, USA). HPLC Column Oven temperature was adjusted to 30 °C. 

For Rg3 measurements, first, 900 µL of ethyl acetate was added to 200 µL samples [38]. Then, it was vortexed for 1 min. The organic phase was then separated from the aqueous phase by centrifugation at 3500× *g* for 3 min. The organic phase was transferred to a clean tube. After evaporation to dryness under vacuum at 40 °C, the residue was reconstituted in 200 µL of methanol: water (95:5 *v*/*v*) and 10 µL of samples and standard solutions of Rg3 (25, 50, 100, 200, and 400 µg/mL) were injected to HPLC. HPLC system was operated isocratically at a flow rate of 0.8 mL min^−1^. The mobile phase consisted of methanol (Merck):10 mM ammonium acetate (Merck) (95:5, *v*/*v*). Peaks were monitored at a wavelength of 203 nm. Rg3 release concentration was quantified by using a standard curve. 

For DOX measurement, the mobile phase consisted of acetonitrile (Merck, Sweden): ammonium hydrogen phosphate aqueous solution (0.01 M) (Sigma) (45:55) at pH 6.2 [39]. Flow rate was set at 0.6 mL/min. A total of 10 µL of all the resuspended released samples and standard solutions of Rg3 (100, 50, 25, 12.5, 6.25, and 3.125 µg/mL) were injected to HPLC. Peaks were monitored at a wavelength of 252 nm and the DOX release concentration was quantified by using a standard curve.

### 2.6. ROS Measurement 

ROS were estimated according to a method described previously [40]. Intracellular ROS were measured based on the intracellular peroxide-dependent oxidation of 2′,7′-dichlorodihydrofluorescein diacetate (DCFH-DA) to form the fluorescent compound, 2′,7′-dichlorofluorescein (DCF). Cells were seeded onto 96-well plates at a density of 2 × 10^4^ cells per well and cultured for 24 h before treatment with GO, GO–Rg3, GO–Rg3–DOX, and DOX for 24 h. Cells treated with H_2_O_2_ (1.7 mg/mL) for 1 h were used as the positive control. The cells were then supplemented with 20 μM of DCFH-DA (Sigma), and incubation continued for 30 min at 37 °C. The cells were rinsed with PBS and trypsinized, and the fluorescence intensity was determined using the CellStream flow cytometer (Luminex, Austin, TX, USA) with excitation at 488 nm and emission C3. Fluorescence intensity was quantified using Luminex data analysis software.

### 2.7. RNA Extraction and Sequencing

Cells were seeded onto 6-well plates at a density of 4 × 10^5^ cells per well and cultured for 24 h before treatment with GO, GO–Rg3, GO–Rg3–DOX, and DOX for 6 h. Medium only was used as the negative control. Three replicates were included from each treatment. After treatment, the cells were washed with PBS buffer three times. Total RNA extraction was performed through RNeasy Mini Kit (Qiagen, Hilden, Germany), and the quality examination was performed by Bioanalyzer (Agilent, Santa Clara, CA, USA) through Agilent RNA 6000 Nano Kit. The quality scale used was Sanger/phred33/Illumina 1.8+. The library was prepared through Illumina TruSeq Stranded mRNA (San Diego, CA, USA). Samples were sequenced on NovaSeq6000 (NovaSeq Control Software 1.7.5/RTA v3.4.4) with a 151nt(Read1)-10nt(Index1)-10nt(Index2)-151nt(Read2) setup using ‘NovaSeqXp’ workflow in ‘S4’ mode flowcell. The Bcl to FastQ conversion was performed using bcl2fastq_v2.20.0.422 from the CASAVA software suite. The raw reads were processed with the NGI RNAseq Pipeline (https://github.com/SciLifeLab/NGI-RNAseq (accessed on 5 November 2021)). The GRCh37 reference genome was used to map the reads (https://support.illumina.com/sequencing/sequencing_software/igenome.html (accessed on 5 November 2021)), and the percentage of uniquely mapped reads was between 79.7–83.6%. Raw read counts were calculated with feature counts. In total, 448,620,449 reads were uniquely mapped on the reference genomes. Samples contained between 8,598,597 to 14,340,446 unique reads. The coverage and quality of RNA sequencing results are summarized in Table S1. The data are accessible through GEO Series accession number GSE185139 (https://www.ncbi.nlm.nih.gov/geo/query/acc.cgi?acc=GSE185139).

### 2.8. Differential Expression (DE) Analysis

Differential gene expression analysis was conducted using DESeq2 (v1.36.0) in R [41] with count values as input. Before differential expression analysis, low-count genes were removed, i.e., only genes with at least 10 counts in at least 3 samples were retained. Volcano plots were then generated using EnhancedVolcano (v1.14.0) (accessed on 26 September 2022) [42] to visualize the results of the differential expression analysis. A gene was considered to be differentially expressed if it had an absolute log-fold change (FC) greater than one (|log2(FC)| > 1) at an adjusted *p*-value (p_adj_) less than 0.01 (p_adj_ < 0.01). All p_adj_ values reported in the study were adjusted to control for the FDR using the Benjamini–Hochberg procedure. 

### 2.9. Gene Set Analysis

Gene set analysis (GSA) was performed using a MATLAB implementation (https://github.com/JonathanRob/GeneSetAnalysisMatlab (accessed on 26 September 2022)) of the R package ‘Piano’ [43] with different gene set collections retrieved from the Molecular Signatures Database (MSigBD) version 7.1 [44], including hallmark [45] and the Gene Ontology (GO) molecular function. The GSA approach we used in this study enables the incorporation of log-fold change directionality (increase or decrease) information for evaluating the significance of gene set enrichment. The enriched gene sets were filtered by p_adj_ < 0.01 for both “non-directional” gene set *p*-values (p_adj.non.dir_) and “distinct directional” *p*-values (p_adj.dist.dir_). The p_adj.dist.dir_ values were calculated for coordinated increases (p_adj,dist-dir-up_) and decreases (p_adj,dist-dir-down_) in expression.

### 2.10. Scanning Electron Microscopy (SEM)

For SEM imaging, cells were seeded onto 12-well plates at a density of 2 × 10^5^ cells per well and incubated for 24 h before treatment with GO, GO–Rg3, GO–Rg3–DOX, and DOX for 24 h. The cells grown in medium without any treatment were used as the control. After treatment, the cells were washed with PBS three times and fixed in 3% glutaraldehyde for 2 h. Finally, the fixed samples were dehydrated with a series of washes with increasing ethanol concentrations (40%, 50%, 60%, 70%, 80%, 90%, and 100%) for 10 min each and then dried for 2 h at room temperature. Before imaging, the dried samples were sputter coated with gold (5 nm). SEM imaging was performed with the Supra 60 VP microscope (Carl Zeiss AG, Jena, Germany).

### 2.11. Transmission Electron Microscopy (TEM) 

For TEM imaging, cells were seeded onto 4-well glass dishes at a density of 2 × 10^5^ cells per well and incubated for 24 h before treatment with GO, GO–Rg3, GO–Rg3–DOX, and DOX for 24 h. The cells grown in medium without any treatment were used as the control. Cells were fixed in modified Karnovsky fixative (2.5% glutaraldehyde, 2% formaldehyde, and 0.02% sodium azide in 0.05 M Na-cacodylate buffer) at 4 °C overnight. The samples were then stained by 1% osmium tetroxide and 1% aqueous uranyl acetate in 0.05 M Na-cacodylate buffer. After staining, samples were subjected to dehydration series with ethanol (30%, 50%, 70%, 85%, 95%, 100%) and infiltration series with epoxy hard plus resin (25%, 50%, 75%, and 100%). Finally, the samples were polymerized in BEEM capsules at 60 °C for 16 h. Ultrathin sections (70 nm) were obtained by a Leica EM UC6 ultramicrotome and imaged by a Thermo Scientific Talos L120C transmission electron microscope.

### 2.12. Confocal Microscopy

For confocal microscopy, 5 × 10^4^ cells per well were grown in 4-well 14 mm glass dishes (MatTek, Ashland, MA, USA) for 24 h and then treated with GO, GO–Rg3, GO–Rg3–DOX, and DOX for 24 h. The cells were washed with PBS buffer several times before observation. An inverted Nikon C2+ confocal microscope with an oil-immersion 60 × 1.4 APO objective was used to acquire images. It is equipped with C2-DUVB GaAsP detectors with variable emission bandpass.

## 3. Results

### 3.1. Rg3 and DOX Were Loaded onto GO to Create GO-Rg3 and GO–Rg3–DOX Conjugates

**Figure 1:**
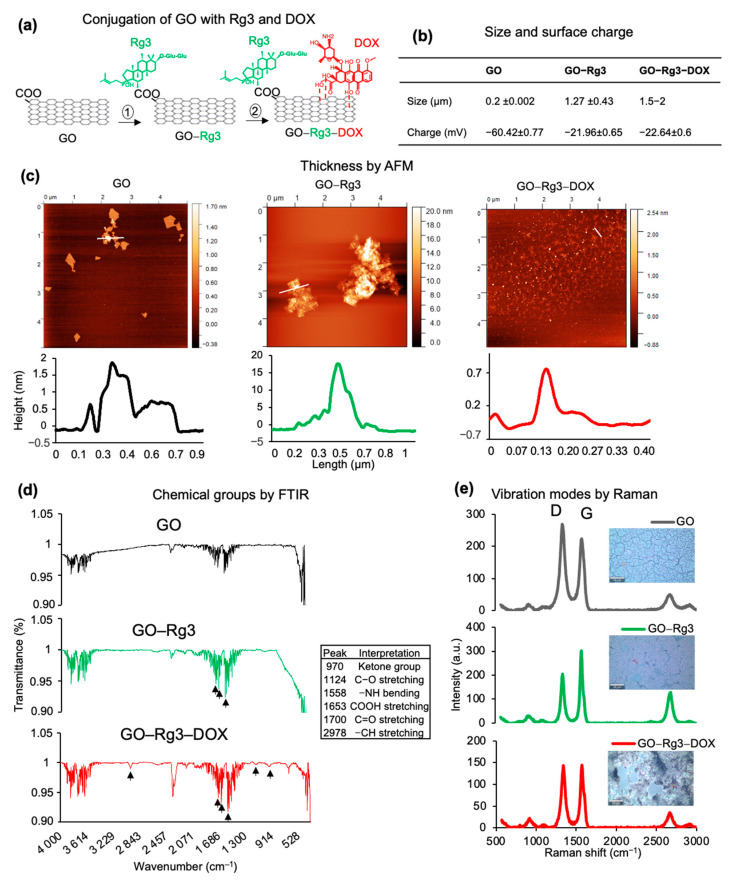
Physical and chemical characterization of GO, GO–Rg3, and GO–Rg3–DOX. (**a**) Schematic depiction of conjugation of GO with Rg3 and DOX. The dotted lines indicate non-covalent binding of DOX to GO. (**b**) Size and surface charge of GO, GO–Rg3, and GO–Rg3–DOX. (**c**) Representative AFM images of GO, GO–Rg3, and GO–Rg3–DOX. Height profile of white lines is shown below each AFM image. (**d**) FTIR analysis of GO, GO–Rg3, and GO–Rg3–DOX. The respective peaks shown by black arrows are listed in the table. (**e**) Raman spectra (left) and bright filed images (right) of GO, GO–Rg3, and GO–Rg3–DOX taken by Raman microscopy.

To produce a biocompatible GO drug carrier, we first conjugated GO nanoflakes with Rg3 and further loaded with DOX (Figure 1a). The GO size was 181.38 ± 2.2 nm (Figure 1b and Figure S1a) on average and the surface charge was −60.42 ± 0.77 mV due to its hydroxyl and carboxyl groups. The flake thickness was in the range of 1–2 nm, as assessed by AFM imaging (Figure 1c). To covalently link Rg3 with GO, concentrated H_2_SO_4_ (98%) was utilized, as described previously [35]. Loading Rg3 to GO increased the average flake size to 1269.75 ± 43.4 nm (Figure 1b and Figure S1a) and flake thickness to above 10 nm; this is most likely due to a negative impact on GO dispersibility in acidic conditions (Figure 1c) [46]. The negative surface charge was decreased to −21.96 ± 0.65 mV upon loading Rg3 due to the esterification of carboxyl groups with Rg3. This is potentially beneficial, since positively charged surfaces are more likely to interact with the high negative charge present on the surface of cancer cells [35]. Finally, GO–Rg3 conjugates were incubated in a solution of DOX (0.1 mg/mL) for drug loading. The alkaline conditions for DOX loading (pH 8) improved the dispersibility of GO–Rg3–DOX flakes and restored the average flake thickness to 1–2 nm (Figure 1c) [46]. The size of the GO–Rg3–DOX flakes increased to 1.5–2 µm, which was out of range for DLS measurement. The size of the particles was instead estimated using AFM (Figure S1b). The DOX loaded onto GO appeared as dots with increased height in AFM (Figure 1c and Figure S1b). This was in line with previously reported edge-adsorption patterns of aromatic compounds on GO [47]. The surface charge was not affected by attachment of DOX and remained at −22.64 ± 0.6 mV. 

In addition to the abovementioned physical parameters, the chemical composition of the drug–GO complexes was characterized using FTIR (Figure 1d) and Raman spectroscopy (Figure 1e). The FTIR spectra of GO–Rg3 and GO–Rg3–DOX showed the C=O stretching vibration for ester groups with a strong peak at around 1700 cm^−1^, which supports esterification reactions occurring between the carboxyl groups of the GO surface and the hydroxyl groups of Rg3 (Figure 1d). The spectrum of pure DOX shows a typical band at 2924 cm^−1^ (corresponding to C-H stretching), and this band is also apparent in the spectra of GO–Rg3–DOX at a slightly shifted wavenumber (2978 cm^−1^). The band at 991 cm^−1^ is characteristic of the ketone groups of DOX, and this was also found in a slightly shifted form (970 cm^−1^) in GO–Rg3–DOX. These results confirm that DOX was successfully loaded onto GO–Rg3. The usual Raman peaks of graphene were detected in GO, GO–Rg3, and GO–Rg3–DOX samples [48]. The G band (~1585 cm^−1^) is a result of in-plane vibrations of sp^2^-bonded carbon atoms, whereas the D band (~1326 cm^−1^) is due to out-of-plane vibrations attributed to the presence of structural defects. GO has a higher D band than pure graphene due to the disruption of sp^2^ bonds by oxidative defects (Figure 1e). The lower D bands in the GO–Rg3 and GO–Rg3–DOX samples confirmed that esterification neutralized these defects and increased the overall order of sp^2^ bonds. For all samples, the minor 2D peak was also identified, confirming the multilayer status of GO flakes, in accordance with the measures’ thickness (Figure 1e) [49]. Taken together, the physical and chemical characterization confirmed the successful loading of Rg3 and DOX onto GO flakes. 

### 3.2. GO Increases ROS Production in Huh7 Cancer Cells

Hepatocellular carcinoma is a leading cause of cancer-related deaths worldwide. The high mortality is mainly due to widespread prevalence and the lack of effective treatment since systemic chemotherapy is ineffective [50]. Considering the challenges in treatment of hepatocellular carcinoma, and the known effect of Rg3 on the induction of apoptosis in liver cancer cells [51], we selected Huh7 hepatocarcinoma cells to test the applicability of our GO–Rg3–DOX platform. 

**Figure 2:**
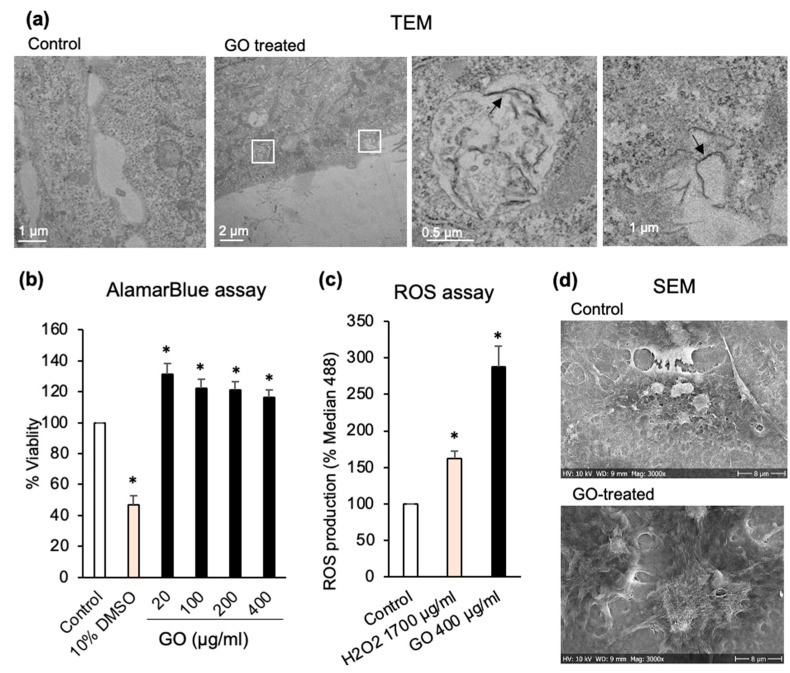
GO alone induces ROS generation in Huh7 cells, which leads to a small decrease of cell viability upon longer exposure. (**a**) TEM images of Huh7 control cells and cells treated with 400 µg/mL of GO for 6 h. Evidence of GO internalization is marked with white boxes, with higher magnification images and black arrows pointing to internalized GO flakes. (**b**) AlamarBlue cell viability assay 24 h after administration of different concentrations of GO. All values are normalized to those obtained for untreated cells (medium alone). The positive control was 10% DMSO. (**c**) ROS production induced by 400 µg/mL of GO after 24 h of treatment, measured at excitation at 488 nm on a flow cytometer based on the formation of the fluorescent compound 2′,7′-dichlorofluorescein (DCF) in presence of ROS. Median 488 values represent DCF fluorescent signal and are normalized to the signal from untreated cells. H2O2 was used as a positive control. (**d**) SEM images of Huh7 cells treated with 400 µg/mL of GO for 24 h. Untreated cells are shown as control. Relevant to panel B and C, data represent the mean ± SE of three independent replicates and it was statistically analyzed and compared with the control (* *p* < 0.05) using Student’s *t* test.

As the first step, we examined the effect of GO alone on Huh7 cells. The cells were treated with GO (400 µg/mL) for 6 h. TEM imaging showed that GO flakes interact with the plasma membrane and get internalized by Huh7 cells (Figure 2a). This was consistent with previous reports, which indicated that GO (297 nm platelets) are effectively internalized by liver cancer cells [52]. Next, Huh7 cells were treated with different concentrations of GO (0, 20, 100, 200, and 400 µg/mL) for 24 h. The alamarBlue assay performed after GO treatments indicated that different concentrations of GO did not reduce the viability of Huh7 cells (Figure 2b). Confocal (data not shown) and SEM images of cells treated with minimal 20 (µg/mL) and maximal (400 µg/mL) concentrations of GO confirmed the cell viability results (Figure 2d). No rounded-up or collapsed cells could be observed in GO-treated samples. Huh7 cells treated with 10% DMSO were included as a control for decreased cell viability. When Huh7 cells were exposed to GO (400 µg/mL) over a prolonged time, cell viability started decreasing after 24 h, and continually decreased to 56.4% after 72 h (Figure S2). Previously, it was demonstrated that GO induces intracellular ROS generation in a concentration and time-dependent manner in the human hepatocellular carcinoma cells and macrophages [53,54]. Our findings suggest that a significant increase of ROS generation can probably account for the reduced viability of Huh7 cells treated with GO over prolonged periods. 

**Figure 3:**
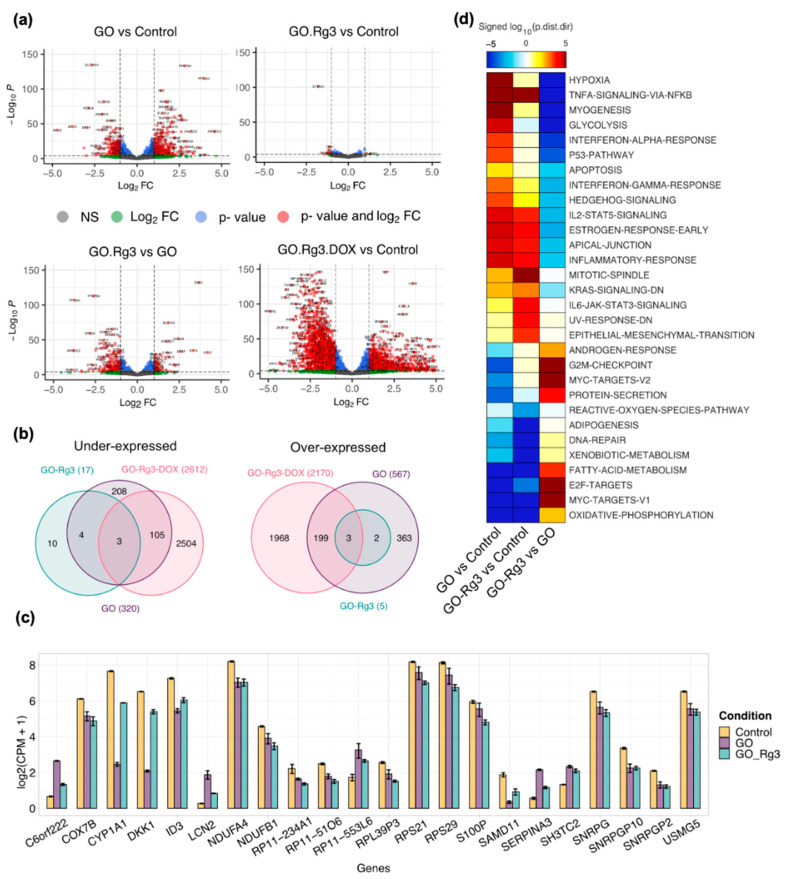
RNA sequencing supports mitigated toxicity of GO by Rg3 conjugation. (**a**) Volcano plots show differentially expressed genes of each treatment. The cut-off for |log2FC| is >1; the cut-off for adjusted *p*-value is 1 × 10^−4^, NS, not significant. (**b**) Venn diagrams showing the number of common and specific overexpressed (right) and under-expressed genes (left) in treatments compared with the control. The cut-off for |log2FC| is >1; the cut-off for adjusted *p*-value is 0.01. (**c**) Barplot of expression of 22 genes differentially expressed genes in GO–Rg3 compared with control. Shown are the log2 transformed count per million (CPM) levels of genes in untreated samples (yellow), treated samples with GO (purple), and treated samples with GO–Rg3 (green). The cut-off for |log2FC| is >1; the cut-off for adjusted *p*-value is 0.01. (**d**) Directional GSA of DE analysis for GO- and GO–Rg3-treated versus control and GO–Rg3- versus GO-treated samples. Only the Hallmark gene set collection is shown here and sets with <10 genes were excluded. The more significant (lower value) of the two directional *p*-values for each gene set is shown in the heatmap as a log10-transformed value. The distinct directional gene set *p*-values (p_adj.dist.dir_) are calculated for coordinated increases (p_adj,dist-dir-up_) and decreases (p_adj,dist-dir-down_) in expression. The value is also “signed,” meaning that gene sets with a more significant decrease than increase (p_adj,dist-dir-down_ < p_adj,dist-dir-up_) are negative; otherwise, they are positive. Only gene sets with a p_adj,dist-dir_ less than 0.01 in at least one comparison are shown.

An RNAseq analysis of Huh7 cells treated with GO (400 µg/mL) revealed a significant number of genes that were differentially expressed compared to the untreated cells: 567 overexpressed and 320 under-expressed genes (Figure 3a,b and Figure S3). Our observation of the higher production of ROS by GO-treated cells was corroborated by the RNAseq analysis (Figure 3d), which showed the downregulation of reactive oxygen species pathways and xenobiotic metabolism genes in Huh7 cells after 6 h of treatment with GO (Figure 3d). Among the ROS pathway gene subset, the glutamate-cysteine ligase modifier subunit (*GCLM*), microsomal glutathione S-transferase 1 (MGST1), glutaredoxin (*GLRX*), glutaredoxin 2 (*GLRX2*), glutathione-disulfide reductase (*GSR*), and glutamate-cysteine ligase catalytic subunit (*GCLC*) were significantly under-expressed in the presence of GO. These genes are involved in the biosynthesis and metabolism of glutathione (GSH), which is one of the main reductive intracellular substances and regulates the level of oxidative stress for maintaining normal cellular function. It was already reported that GO could oxidize GSH to GSSG, leading to the formation of reduced GO. Hence, GSH depletion could affect the intracellular oxidative/reductive balance, leading to increased levels of ROS [55]; this is in line with our observations (Figure 2c), and the cells exposed to GO were not able to scavenge ROS, thereby leading to accumulation. 

Stimulating factor 1 (*CSF1*) complement C5a receptor 1 (*C5AR1*) is also overexpressed by GO. C5AR1 is a receptor for the chemotactic and inflammatory peptide, anaphylatoxin C5a. The C5AR1 receptor activation stimulates chemotaxis, granule enzyme release, intracellular calcium release, and superoxide anion production [56]. Hence, GO might induce superoxide anion production due to the increased expression of *C5AR1*, which is followed by overexpression of superoxide dismutase 2 (*SOD2*) to destroy superoxide anion radicals in GO-treated cells, as is shown in the ROS pathway genes subset in Figure S4. 

The same analysis revealed the downregulation of the oxidative phosphorylation pathway upon GO treatment (Figure 3d). Specifically, NADH:ubiquinone oxidoreductase (NDUF) genes (*NDUFB6*, *NDUFA4*, *NDUFB5*, *NDUFS4*, *NDUFS1*, *NDUFA5*, *NDUFA8*, *NDUFAB1*, *NDUFA6*, *NDUFA2*, *NDUFB3*, *NDUFB1*, and *NDUFV2*) involved in the function of the mitochondrial membrane respiratory chain NADH dehydrogenase (Complex I) were significantly under-expressed in the presence of GO (Figure S4). This is consistent with a previous report that showed that GO attenuates the expression of genes associated with the oxidative phosphorylation complexes, such as *NDUFA1*, *NDUFB3,* and *NDUFS4* in glioblastoma U87 cell line [57]. The electron transport chain complex I transfers electrons from NADH to ubiquinone. Furthermore, the decreased activity of complex I could reduce the growth and induce cell death via oxidative stress [58]. Similar to previous findings [58], cytochrome oxidase c (COX) genes, including *COX7B, COX17, COX7A2L, COX6C, COX7C,* and *COX7A2,* were also under-expressed in the GO-treated samples (Figure S4). Cytochrome c oxidase (COX) is the terminal component of the mitochondrial respiratory chain and catalyzes the electron transfer from reduced cytochrome c to oxygen. 

How does GO influence ROS accumulation? There are several plausible hypotheses supported by the available data. GO can act as an electron donor, supplying electrons to complexes I and II of the electron transport chain. This would accelerate ROS generation as a byproduct of mitochondrial respiration, as reported by Zhang et al. [59]. GO may also affect mitochondrial functioning, modulate the expression of genes involved in mitochondrial activity (e.g., oxidative phosphorylation), and thereby impact ROS generation by mitochondria. However, based on our results, the reduced expression of glutathione biosynthesis genes and the absence of ROS scavengers cumulatively lead to ROS accumulation. 

Among the genes involved in the inflammatory response, inducible T cell co-stimulator ligand (*ICOSLG*), colony stimulating factor 3 receptor (*CSF3R*), and colony stimulating factor 1 (*CSF1*) were significantly overexpressed by GO (Figure S4). *ICOSLG* promotes T cell immune responses while *CSF3R* and *CSF1* are cytokines involved in the differentiation of granulocytes and macrophages. Thus, these results suggest that GO might induce an immune response in cancerous cells.

In addition to ROS metabolism and oxidative phosphorylation, our RNAseq analysis revealed the decreased expression of BH3 interacting domain death agonist (*BID*) and caspase 3 (*CASP3*) in GO-treated cells (Figure S4). BID is the sensor and transducer of apoptotic signals, and it allows the release of cytochrome c. Cytochrome c serves as a platform for the activation of caspase mediated apoptosis. The under-expression of both *BID* and *CASP3* by GO treatment is consistent with the alamarBlue assay results, which showed no apoptosis at 6, 12, and 24 h after the treatment (Figure S2). 

The IL-6/JAK/STAT3 pathway plays a key role in the growth and development of many human cancers [60]. Thus, we also looked at the differentially expressed genes in the IL6-JAK-STAT3 signaling pathway genes subset. GO induced the overexpression of *IL2RG*, *IL17RA*, *IL7*, and *IL4R* genes. Among these, interleukin 17 receptor A (IL17RA) is the receptor for IL17A that is the major proinflammatory cytokine secreted by activated T-lymphocytes. In fact, IL-17 communicates with JAK-STAT family signaling, particularly STAT3 [61]. Furthermore, IL-17 showed a positive association with vascular endothelial growth factor (*VEGF*) expression and signaling [62], which is similar to our observations about the overexpression of VEGF by GO, as shown in the angiogenesis genes subset in Figure S4. STAT3, a member of the STAT protein family, is a signal transducer in the cytoplasm and a transcription activator in the nucleus, and it is activated by cytokines and growth factors. VEGF can also induce cellular processes that are common to many growth factor receptors, including cell migration, expansion, development, and proliferation. In fact, the activation of both of these pathways is indicative of an increase in cell proliferation after exposure to GO (Figure 2b), which is similar to the previous report about the cell proliferative effect of pristine graphene [61]. This can also be supported by increased cell proliferation and growth in numerous cell lines (MCF-7, HepG2, A549, and HeLa cells) treated with pristine graphene [63].

In our analysis, *CYP1A1* and *CYP39A1* genes were strongly under-expressed by GO (Figure 3c and Figure S4). *CYP1A1* and *CYP39A1* genes encode members of the cytochrome P450 superfamily of enzymes, which catalyze reactions involved in drug metabolism. It was previously shown that GO interferes with drug metabolism/detoxification in the body at the level of phase I cytochrome-P450 system by the inhibition of gene expression and metabolic activity [64]. This influence of GO is clinically relevant, since altered drug metabolism can significantly contribute to the variability of drug responses and be associated with an increased risk of adverse effects along with altered detoxification.

Overall, it can be concluded that the GO carrier with an average size of 180 nm can be internalized by Huh7 cells, wherein they induce ROS accumulation, repression of genes associated with oxidative phosphorylation, and result in diminished cell viability starting from 48 h after treatment (Figure S2). All together, these results substantiate a certain level of toxicity of GO. This is a potential drawback for GO as a drug carrier, and we next sought to remedy the issue.

### 3.3. Conjugation of GO with Rg3 Mitigates ROS Production Induced by the Nanocarrier

**Figure 4:**
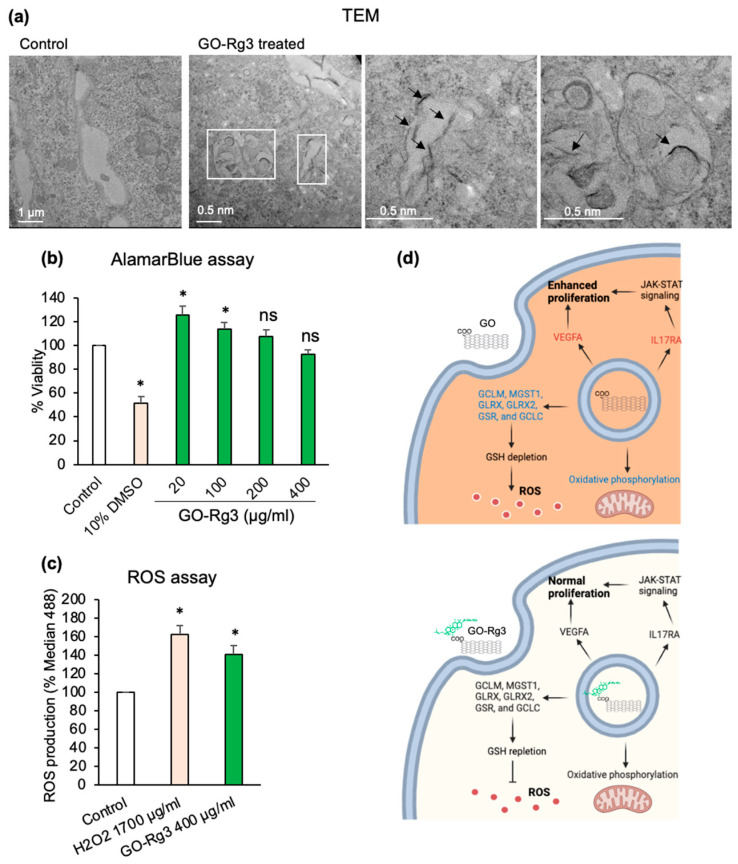
Conjugation of Rg3 with GO reduces oxidative stress induced by the nanocarrier in Huh7 cells. (**a**) TEM images of Huh7 control cells and cells treated with 400 µg/mL GO for 6 h. Evidence of GO internalization is marked with white boxes, with higher magnification images and black arrows pointing to internalized GO flakes. (**b**) AlamarBlue cell viability assay of Huh7 cells treated with GO–Rg3 for 24 h. GO–Rg3 was prepared using the 0.5 mg/mL solution of Rg3 for loading. Different concentrations of GO–Rg3 were administered. All values were normalized to those obtained from untreated cells (medium only). The positive control was 10% DMSO. (**c**) ROS production induced by 24 h exposure of Huh7 cells to 400 µg/mL GO–Rg3. ROS was measured at excitation at 488 nm on a flow cytometer based on the formation of the fluorescent compound 2′,7′-dichlorofluorescein (DCF) in presence of ROS. Median 488 values represent DCF fluorescent signal and are normalized to the signal from untreated cells. H_2_O_2_ was used as a positive control. (**d**) Schematic model of GO (Top) and GO–Rg3 (Down) internalization into Huh7 cells and downstream effects. (Top) GO internalized through endocytosis, under-expressed oxidative phosphorylation, and glutathione-biosynthesis-related genes, leading ROS accumulation. GO enhances cell proliferation through upregulation and activation of JAK-STAT signaling and VEGF. (Down) GO–Rg3 mitigates toxicity of GO by restoring the expression level of oxidative phosphorylation and glutathione biosynthesis genes, leading to reduced ROS production. GO–Rg3 moderates the expression of JAK-STAT signaling and VEGF, leading to normal cell proliferation. Red- and blue-colored genes were over- and under-expressed, respectively, while black means unchanged expression. Relevant to panel B and C, data represent the mean ± SE of three independent replicates and it was statistically analyzed and compared with the control (*: *p* < 0.05, ns: not significant) using Student’s *t* test.

Considering that Rg3 is known to increase the efficacy of DOX treatments while reducing the side effects [30,31,32], and is known to have antioxidative effects [65], which could help with GO-induced ROS production, we decided to conjugate our GO nanocarrier platform with Rg3 (Figure 4a–d). To identify optimal loading conditions, we used two different starting concentrations of Rg3: 0.5 and 1 mg/mL, which resulted in 49% and 40% of total Rg3 loaded onto GO, respectively (Figure S5a). Since neither of the two resulting GO–Rg3 conjugates negatively affected Huh7 cell viability (Figure 4b and Figure S5b), the sample loaded with the lower concentration of Rg3 (0.5 mg/mL, 49% of Rg3 loaded) was used as our standard GO–Rg3 preparation for all subsequent studies. Of note, conjugation of GO with Rg3 did not affect the internalization of the nanocarrier by the Huh7 cells, and GO–Rg3 could enter via endocytosis, as evidenced by TEM imaging (Figure 4a). Using this preparation of GO–Rg3, we assessed the impact on Huh7 cells with confocal microscopy, and no rounded-up or collapsed cells were observed (Figure S5c). ROS measurements (Figure 4c) showed that the conjugation of GO with Rg3 strongly reduced the ROS levels induced in Huh7 cells by the nanocarrier (compare to Figure 2c). 

The beneficial effects of Rg3 were also confirmed by RNAseq results (Figure 3). As shown in Figure 3a, very few genes were differentially expressed in GO–Rg3-treated cells compared to untreated cells (22 genes) (Figure 3b,c). Among those, only 12 differentially expressed genes overlapped with differentially expressed genes of GO-treated cells (Figure 3b). Rg3 conjugation cancelled the effect of GO on genes involved in the reactive oxygen species pathway and xenobiotic metabolism genes (Figure 3d). For example, cytochrome P450 enzymes are involved in drug metabolism, and CYP1A1 and CYP39A1 are highly under-expressed by GO. In GO–Rg3-treated cells, the expression level of these genes was close to normal; this suggests that Rg3 functionalization cancels the gene-expression-altering effect of GO alone. Similar results were obtained for other classes of genes. For example, as stated in the previous section, glutathione-biosynthesis-related genes were significantly under-expressed in GO-treated cells, thereby leading to GSH depletion and increased levels of ROS (Figure S4 and Figure 2c). Functionalization with Rg3 reversed the effect of GO on the expression of glutathione-biosynthesis-related genes, including *GCLM*, *MGST1*, *GLRX*, *GLRX2*, *GSR*, and *GCLC* (Figure S4), thereby preventing ROS accumulation (Figure 4c). This result is consistent with a previous report claiming that Rg3 protects hepatocytes against toxic metabolites produced from widely used analgesic and antipyretic drugs including benzo[α]pyrene and acetaminophen. GSH repletion and coordinated gene regulation of GSH synthesis was proposed as the mechanism behind the beneficial effect of Rg3 [66].

Conjugation of Rg3 to GO restored the expression of inflammatory response genes, including *ICOSLG*, *CSF3R*, *CSF1*, and *C5AR1,* which were overexpressed in GO-treated cells to the normal levels (Figure S4). 

Furthermore, treatment of cells with the conjugated form of GO–Rg3 at 400 mg/mL did not induce IL17RA and VEGF gene expression neither cell proliferation (Figure S4 and Figure 4), which are explained as unwanted side effects of GO carrier. 

Overall, Rg3 conjugation reduced the observed levels of GO cytotoxicity by diminishing ROS-induced stress and normal cell proliferation (Figure 4d). Thus, conjugation of Rg3 with GO makes GO–Rg3 a proper carrier with less toxicity compared to GO, which will drive the drug to the target cells with minimal toxicity. 

### 3.4. Effect of GO–Rg3–DOX on Hepatocarcinoma and Breast Cancer Cells

#### 3.4.1. pH Dependent Release of DOX from GO–Rg3–DOX 

The pH of blood and tissue is tightly controlled around pH 7.4 under normal physiological conditions. However, a local pH range from 5.5 to 7.0 is not uncommon in diseased tissues, such as the tumor microenvironment [67,68]. According to previous studies, DOX loading onto GO could be attributed to simple π-stacking [69] and its release from GO is enhanced at low pH [37]. The daunosamine group of DOX can be protonated, and thus its solubility is increased in acidic conditions, favoring DOX release from GO [70]. In our study, GO is negatively charged while Rg3 functionalization decreased the negative charge of GO, which might be due to the esterification of carboxyl groups with Rg3 (Figure 1b). However, the addition of DOX to GO–Rg3 did not change the surface charge of GO–Rg3. We expect that the reduced negative charge of GO–Rg3–DOX compared to GO is beneficial for interacting with the high negative charges present on the surface of cancer cells. This is similar to previous studies on GO functionalized with cationic antibiotic and its electrostatic interaction with negatively charged bacteria to facilitate bacterial trap-and-kill [71]. The DOX is possibly bound with GO–Rg3 through hydrogen bonding, which can be weakened at an acidic pH. This feature enhances targeted drug delivery since hydrophobic DOX, which is bound with hydrophilic GO, can be released specifically in the acidic tumor microenvironment or, upon internalization by Huh7 cells, inside the acidic endolysosomes. To examine the release of Rg3 and DOX from GO–Rg3–DOX, we placed a GO–Rg3–DOX solution (100 µg/mL) into a dialysis bag and incubated it over 12 days at two different pH values: 5.5 and 7.4 (Figure S6). At regular intervals, the release of Rg3 and DOX was measured by HPLC. Rg3 showed a steady release profile, independent of the pH value (Figure S6b). By contrast, the DOX release showed strong pH dependence, with almost no release at pH 7.4 and a steady release at pH 5.5 (Figure S6c). This finding was in accord with a previous report claiming that DOX was not released from GO at pH 7.4 [37]. Therefore, we concluded that pH 5.5, corresponding to an acidic tumor microenvironment and endolysosomal lumen inside targeted cells, would be the optimal conditions for DOX release from GO–Rg3–DOX. The retention of DOX on the GO carrier at a normal physiological pH of 7.4 means that the carrier would not release any DOX in the bloodstream or in non-targeted tissues, which would dramatically reduce the systemic side effects of this anticancer drug. 

#### 3.4.2. DOX Delivered in the Form of GO–Rg3–DOX Is More Effective Than Free DOX 

**Figure 5:**
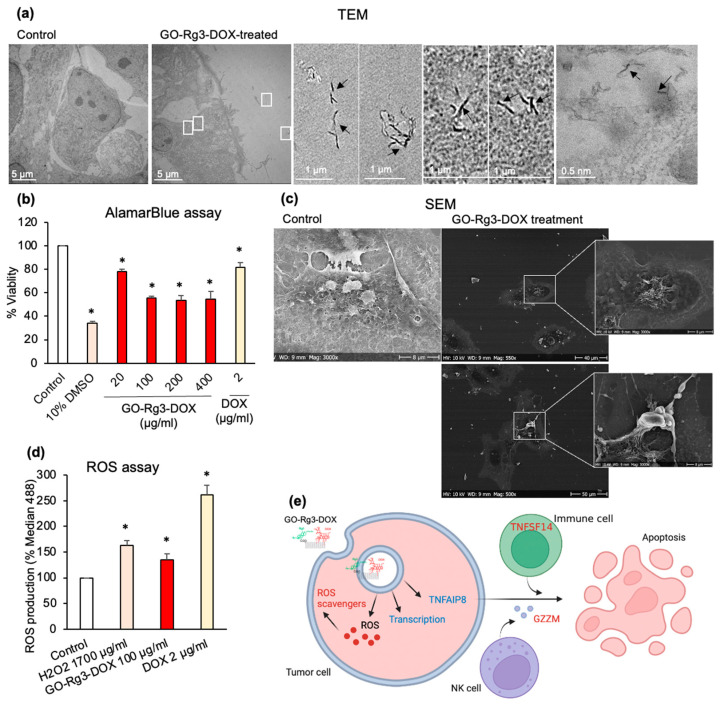
Cytotoxicity of GO–Rg3–DOX against Huh7 cells. (**a**) TEM images of Huh7 cells treated with 100 µg/mL GO–Rg3–DOX for 6 h compared to untreated cells. Evidence of extracellular GO and GO internalization are marked with white boxes, with higher magnification images and black arrows pointing to GO flakes. (**b**) AlamarBlue cell viability assay 24 h after administration of different concentrations of GO–Rg3–DOX. All values were normalized to those obtained from untreated cells (medium only). The positive controls were 10% DMSO and 2 µg/mL DOX. (**c**) SEM images of Huh7 cells treated with GO–Rg3–DOX (400 µg/mL) for 24 h, compared to untreated cells as control. (**d**) ROS production induced by GO–Rg3–DOX 24 h after administration, measured at excitation at 488 nm on a flow cytometer based on the formation of the fluorescent compound 2′,7′-dichlorofluorescein (DCF) in presence of ROS. Median 488 values represent DCF fluorescent signal and are normalized to the signal from untreated cells. H2O2 was used as a positive control. (**e**) Schematic representation of GO–Rg3–DOX internalization and downstream effects. Upon GO–Rg3–DOX treatment, transcription-related genes were under-expressed, while ROS-scavengers-related genes were overexpressed to detoxify the cell. Under-expression of TNFAIP8 and overexpression of GZMM and TNFSF14 induce cancer cell apoptosis. Red- and blue-colored genes were over- and under-expressed, respectively. Relevant to panel B and E, data represent the mean ± SE of three independent replicates and it was statistically analyzed and compared with the control (* *p* < 0.05) using Student’s *t* test.

Next, we set out to compare the effectiveness of DOX delivered by GO–Rg3–DOX versus free DOX against Huh7 cells. A total of 100 µg/mL of GO–Rg3–DOX carrier was found to release between 1 and 1.6 µg/mL of DOX within 24 h, depending on pH (Figure S6c). Hence, a solution of 2 µg/mL of free DOX was selected as a benchmark for comparison. Our TEM analysis confirmed that GO–Rg3–DOX could enter the Huh7 cells (Figure 5a). Huh7 cells were treated with different concentrations of GO–Rg3–DOX (0, 20, 100, 200, and 400 µg/mL) and 2 µg/mL of DOX for 24 h. GO–Rg3–DOX significantly reduced the viability of Huh7 cells at all tested concentrations. Dead cells in GO–Rg3–DOX-treated samples were also observed by SEM imaging (Figure 5c). Importantly, the anticancer effect of GO–Rg3–DOX (44–47% dead cells at carrier concentrations of 100–400 µg/mL) was consistently at least two-fold higher than DOX alone at benchmark concentration (18.5% dead cells) (Figure 5b). We also expanded the range of DOX treatment to concentrations higher than the selected benchmark, which were 4–8 µg/mL—2–4 times higher than benchmark (Figure S7a). However, there were no significant changes in the viability of cells treated with even higher concentrations of free DOX. The result was also compared with the IC50 concentration of free DOX treatment against Huh7 cells from other studies (Figure S7c). Other studies reported an IC50 of 5–10 µg/mL for free DOX while GO–Rg3–DOX could effectively achieve 44–47% dead cells by releasing less than 2 µg/mL. Even the enhanced toxicity of GO–Rg3–DOX compared to free DOX might be in line with the previous finding that Rg3 can enhance the anticancer effects of DOX [50]. Interestingly, the cell viability assay showed no significant difference in the concentration range of 100–400 µg/mL of GO–Rg3–DOX (Figure 5b). 

Due to the saturation effect, 100 µg/mL of GO–Rg3–DOX was selected as the working concentration to further investigate the effect of GO–Rg3–DOX upon cell viability. The impact on cell viability was examined over a longer time course, showing 81% dead cells after 72 h of treatment (Figure S2). Interestingly, while GO–Rg3–DOX was twice as effective as free DOX at killing cancer cells, it induces less than half of ROS formation compared to DOX (Figure 5d). Therefore, it seems plausible to conclude that Rg3 enhanced the biocompatibility of our GO–Rg3–DOX nanocarrier by reducing the amount of ROS, thereby lowering the potential for creating side effects without diminishing DOX cytotoxicity to cancer cells. To test this hypothesis, we sought to clarify the mechanism of GO–Rg3–DOX cytotoxicity to Huh7 cells, which was addressed by a gene expression data analysis of treated cells.

GO–Rg3–DOX treatment affected the expression of many genes in Huh7 cells (Figure 3a). A total of 2170 genes were overexpressed, and 2612 genes were repressed by the treatment, compared to the untreated cells (FDR cutoff 0.01 and minimum fold change 1) (Figure 3b). Among differentially expressed genes, 310 genes overlapped with the genes that were differentially expressed in GO-treated cells, and only six genes overlapped with the genes that were differentially expressed in GO–Rg3-treated cells. 

Directional GSA of DE analysis of GO-, GO–Rg3-, and GO–Rg3–DOX-treated versus control untreated samples showed that the transcription and transcription regulatory genes were significantly repressed by GO–Rg3–DOX (Figure S8A,B). Many transcription regulatory genes with functioning in development and differentiation were under-expressed (Table S1). Thus, the decreased expression of these genes by GO–Rg3–DOX could clearly be expected to affect the transcription of developmental genes. 

In cells treated with GO–Rg3–DOX, the genes encoding the proteins with antioxidant activity were overexpressed (Figure S8 and Table S1). In fact, the critical apoptotic trigger of DOX is the oxidative DNA damage by the DOX-induced direct H_2_O_2_ generation [72]. Thus, the induction of these antioxidative genes confirms a strong therapeutic effect of DOX released from GO–Rg3–DOX. 

GO–Rg3–DOX provoked the overexpression of protease genes with the apoptotic function, such as granzyme M (*GZMM*), Caspase-10 (*CASP10*), and hyaluronan binding protein 2 (*HABP2*) (Figure S8 and Table S1). Furthermore, we could also find differentially expressed genes with the apoptotic function in the peptidase regulatory gene subset, such as BCL2 associated agonist of cell death (*BAD*) and TNF superfamily member 14 (*TNFSF14*), which were overexpressed, and TNF alpha induced protein 8 (*TNFAIP8*), which was under-expressed in GO–Rg3–DOX-treated cells. BAD promotes cell death, and TNFSF14 protein stimulates the proliferation of T cells and trigger apoptosis of tumor cells—while TNFAIP8 acts as the negative mediator of apoptosis. 

Regarding immune-response-related genes, azurocidin 1 (*AZU1*), signal peptide peptidase like 2B (*SPPL2B*), and cathepsin W (*CTSW*) were overexpressed in GO–Rg3–DOX-treated cells (Figure S8B). Hence, these results suggest that GO–Rg3–DOX might induce an immune response in cancerous cells.

In summary, GO–Rg3–DOX is twice as effective as free DOX while inducing less than half of the ROS. Furthermore, besides cancer cell death induced by ROS, it effectively represses the transcription of developmental genes and triggers tumor apoptosis.

#### 3.4.3. GO–Rg3–DOX Is Cytotoxic against Human MDA-MB-231 Breast Cancer Cells

**Figure 6:**
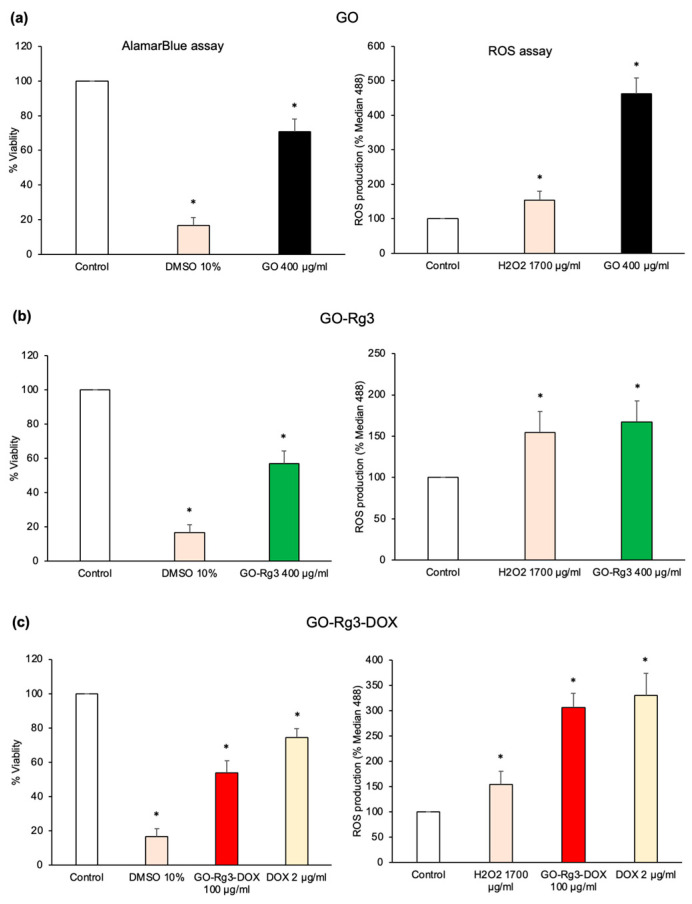
Rg3 and DOX cytotoxicity in human breast cancer MDA-MB-231 cells. (**a**) AlamarBlue assay 24 h after GO administration. All values were normalized to those obtained from untreated cells (medium only). The positive control was 10% DMSO. (**b**) AlamarBlue assay 24 h after GO–Rg3 administration. All values were normalized to those obtained from untreated cells (medium only). The positive control was 10% DMSO. (**c**) AlamarBlue assay 24 h after GO–Rg3–DOX administration. All values were normalized to those obtained from untreated cells (medium only). The positive control was 10% DMSO. In all panels, ROS production 24 h after administration is shown on the right side, measured at excitation at 488 nm on a flow cytometer based on the formation of the fluorescent compound 2′,7′-dichlorofluorescein (DCF) in presence of ROS. Median 488 values represent DCF fluorescent signal and are normalized to the signal from untreated cells. H2O2 was used as a positive control. Relevant to all panels, data represent the mean ± SE of three independent replicates and it was statistically analyzed and compared with the control (* *p* < 0.05) using Student’s *t* test.

DOX is currently the most effective chemotherapeutic drug used to treat breast cancer. In order to test the potential of our GO–Rg3–DOX platform for broader cancer therapy, we tested it against another type of cancer cell, the MDA-MB-231 breast cancer cell line. This cell line is resistant to DOX and free DOX treatment could only reduce cell viability by 22% at 8 µg/mL, which is 4 times higher than the benchmark concentration (Figure S7b). Based on the previous assays with Huh7 cells, we have selected 400 µg/mL of GO, 400 µg/mL of GO–Rg3, 100 µg/mL of GO–Rg3–DOX, and 2 µg/mL of free DOX as the standard working concentrations (Figure 6). After 24 h of treatment, it was observed that DOX alone could only kill 25.5% of the cells (Figure 6c). By contrast, GO–Rg3 and GO–Rg3–DOX eliminated 43% and 46.3% of the cells, respectively (Figure 6b,c). Our ROS measurements suggested a similar scenario as the one seen with Huh7 cells. DOX-induced cardiotoxic side effects are known to involve ROS generation [34]. In the MDA-MB-231 cells, Rg3 mitigated GO-induced ROS generation (Figure 6b). Therefore, it can be concluded that the novel DOX conjugate (GO–Rg3–DOX) is an efficient anticancer agent (46.3% cytotoxicity) against breast cancer cells. After 24 h of treatment, its killing efficiency was significantly higher than the current benchmark study, where the cytotoxicity effect of combined free Rg3 and free DOX was reported against the same cell line, but with double the exposure (48 h) [34]. In addition to GO–Rg3–DOX being effective, its Rg3 component reduced ROS generation, which is expected to reduce the side effects on non-cancerous tissues. 

## 4. Conclusions

Based on the results reported in this study, we propose that our pH-responsive DOX carrier, GO–Rg3–DOX, could present a promising new venue for efficient delivery of chemotherapy to cancerous tissues with minimized toxicity of drug carrier. This combination of a GO nanocarrier, Rg3, and DOX possesses several key properties that go beyond state-of-the-art in current drug delivery approaches. Firstly, GO–Rg3–DOX is more effective in terms of cytotoxicity towards Huh7 and MDA-MB-231 cells compared to all the benchmark studies in the literature, and can help to reduce the quantity of chemotherapy drugs administered, thereby reducing the side effects and cost of treatment. Secondly, GO–Rg3–DOX releases the loaded drugs only in the acidic environment, which ensures specific release in the acidic environment of cancerous tissues, and, upon endocytosis, in endolysosomes of targeted cells. This feature could be key for targeted delivery and minimizing systemic side effects. Finally, the presence of Rg3 in the platform significantly reduces ROS formation and moderates cell proliferation, which are otherwise stimulated by GO alone. These Rg3 effects show promise for mitigating side effects that are commonly related to unwanted ROS damage to healthy tissues. The biocompatibility and effectiveness of this novel drug delivery system need to be further investigated in different mammalian cell lines, but current results support its relevance for further testing in animal models. If GO–Rg3–DOX could be combined with a receptor-mediated targeted delivery system, it has potential to become a powerful tool for chemotherapy.

## Data Availability

Raw RNA sequencing data and raw read counts are accessible through GEO Series accession number GSE185139 (https://www.ncbi.nlm.nih.gov/geo/query/acc.cgi?acc=GSE185139).

## Supplementary Materials

The following supporting information can be downloaded at: https://www.mdpi.com/article/10.3390/pharmaceutics15020391/s1. Figure S1. (a) Dynamic light scattering (DLS) size distribution curve of GO and GO-Rg3 in combination with normalized intensity correlation function. (b) Atomic force microscopy (AFM) image of GO-Rg3-DOX. Height profile of white line is shown below each AFM image. Figure S2. GO and GO-Rg3-DOX are toxic for Huh7 cells during prolonged treatments. AlamarBlue cell viability assay with Huh7 cells treated with 400 µg/ml GO and 100 µg/ml GO-Rg3-DOX, at different time points. All values were normalized to those obtained with untreated cells (grown in medium only). 10% DMSO was used as a positive control. Figure S3. PCA plot of gene expression of Huh7 cells treated with GO, GO-Rg3, and GO-Rg3-DOX compared to untreated cells as the control. (a) Log-CPM of all genes were used in PCA analysis, (b) Log-CPM of only associated genes with metabolism were used. Points are colored for different treatments. Figure S4. RNA sequencing supports mitigated toxicity of GO by Rg3 conjugation. Significantly changed genes in gene sets associated with the xenobiotic metabolism, angiogenesis, reactive oxygen species, IL6-JAK-STAT3 signaling, oxidative phosphorylation, inflammatory response, and apoptosis, are shown in the heatmaps, colored by log_2_FC of the genes in GO-treated and GO-Rg3-treated compared with untreated samples, and also GO-Rg3-treated compared with GO-treated samples. Log fold-change directionality (increase or decrease) information was incorporated with log_10_ (p_adj_) for representing the significance of differential expressed genes in each gene set. Figure S5. (a) Loading capacity of GO for Rg3. GO was conjugated with Rg3 at concentrations 0.5 and 1 mg/ml. The remaining material after GO loading with Rg3 was used for HPLC analysis. (b) AlamarBlue cell viability assay of Huh7 cells treated with GO-Rg3 for 24 h. GO-Rg3 was prepared using the 1 mg/ml solution of Rg3 for loading. Different concentrations of GO-Rg3 were administered. All values were normalized to those obtained from untreated cells (medium only). 10% DMSO was used as a positive control. Data represent the mean ± SE of three independent replicates and it was statistically analyzed and compared with the control (*: *p* < 0.05, ns: not significant) using Student’s *t* test. (c) Confocal imaging of Huh7 cells after treatment with our standard preparation of GO-Rg3. Cell were incubated for 24 h in a medium containing 20 µg/ml GO-Rg3 and were subsequently washed with PBS before imaging. 10% DMSO was used as a positive control. Figure S6. pH-dependent DOX and Rg3 release from GO-Rg3-DOX nanocarriers. (a) Schematic outline of the experimental set-up. (b) Cumulative Rg3 release over twelve days at pH 5.5 and pH 7.4. (c) Cumulative DOX release over six days at pH 5.5 and pH 7.4. The Rg3 and DOX release to PBS buffer was measured by HPLC. Figure S7. AlamarBlue viability assay of (a) Huh7 and (b) MDA-MB-231 cells treated with different concentrations of DOX for 24 h. All values were normalized to those obtained from untreated cells (medium only). 10% DMSO was used as a positive control. Data represent the mean ±SE of three independent replicates. (c) IC50 of DOX against Huh7 cells after 24 h treatment from other studies (black) and our study (red). Figure S8. Effect of DOX delivered by GO-Rg3-DOX. (A) Results of the directional GSA of DE analysis for GO, GO-Rg3, and GO-Rg3-DOX treated versus control samples. Only the molecular function (GO_terms) gene set collection retrieved from downloaded MSigBD database is shown here, and sets with <10 genes were excluded. The more significant (lower value) of the two directional *p* values for each gene set is shown in the heatmap as a log_10_-transformed value. The distinct directional gene set *p* values (p_adj.dist.dir_) are calculated for coordinated increases (p_adj,dist-dir-up_) and decreases (p_adj,dist-dir-down_) in expression. The value is also “signed,” meaning that gene sets with a more significant decrease than increase (p_adj,dist-dir-down_ < p_adj,dist-dir-up_) are negative; otherwise, they are positive. Only gene sets with a p_adj,dist-dir_ less than 1 × 10^−6^ in at least one of the comparisons are shown. (B) Significantly changed genes in gene sets associated with double strand DNA binding, ion transmembrane transporter activity, endopeptidase activity, antioxidant activity, and peptidase regulator activity are shown in the heatmap, colored by log_2_FC of the genes in GO, GO-Rg3, and GO-Rg3-DOX treated samples compared with untreated samples, and also GO-Rg3 compared with GO treated samples. The cut-off for all gene sets (except ion transmembrane transporter activity |log_2_FC| is >2) |log_2_FC| is >1. Log fold-change directionality (increase or decrease) information was incorporated with log_10_ (p_adj_) for representing the significance of differential expressed genes in each gene set. Genes associated with these gene sets were extracted from molecular function gene set. Table S1. Coverage and quality of RNA sequencing (RNA-seq) results of samples used in this study. GRCh37 was used as the reference genome. Table S2. Selected differentially expressed genes affected by GO-Rg3-DOX treatment compared with non-treated samples in gene sets associated with double strand DNA binding, endopeptidase activity, antioxidant activity, and peptidase regulator activity. References [73,74,75] are cited in the supplementary materials.
