# Supplementary material for: Ginsenoside Rg3 Reduces the Toxicity of Graphene Oxide Used for pH-Responsive Delivery of Doxorubicin to Liver and Breast Cancer Cells
Source: Pharmaceutics. 2023 Jan 24;15(2):391. doi: 10.3390/pharmaceutics15020391 (PMC9965446; doi:10.3390/pharmaceutics15020391)
Supplement: Supplementary file 1 [file pharmaceutics-15-00391-s001.zip › pharmaceutics-2133867-supplementary.pdf]

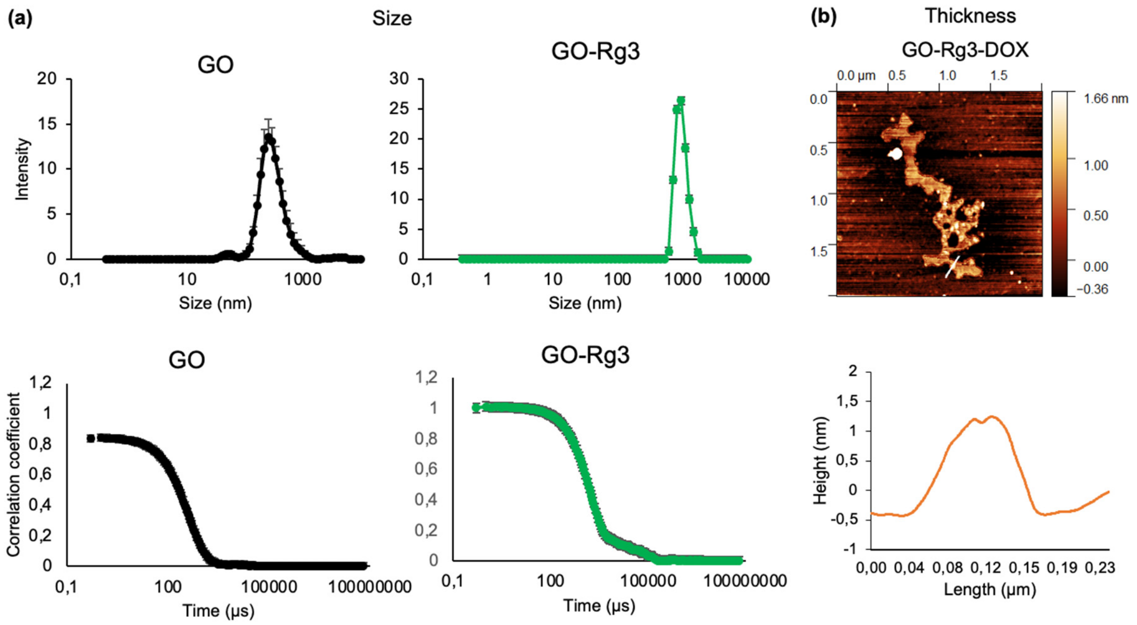

Figure S1. (**a**) Dynamic light scattering (DLS) size distribution curve of GO and GO-Rg3 in combination with normalized intensity correlation function. (**b**) Atomic force microscopy (AFM) image of GO-Rg3-DOX. Height profile of white line is shown below each AFM image.

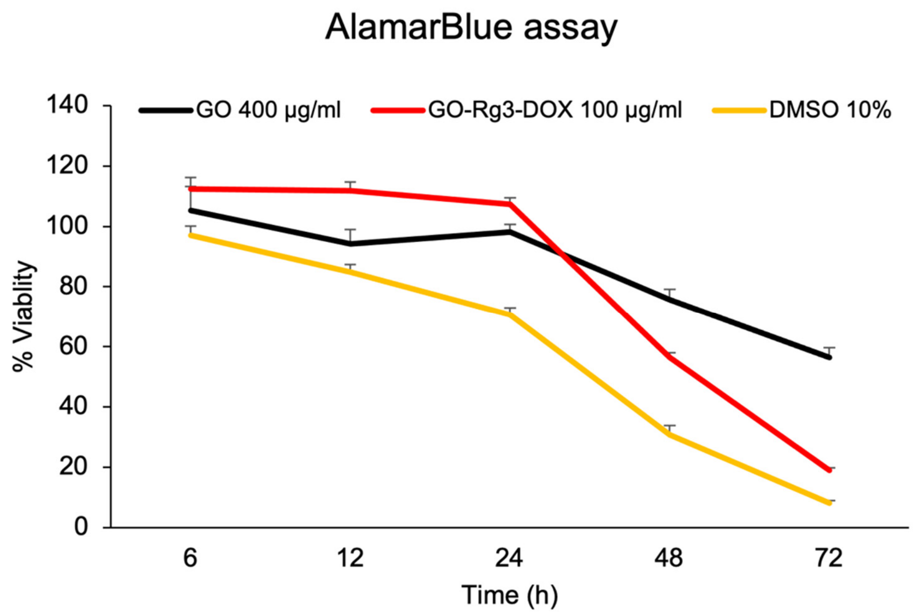

Figure S2. GO and GO-Rg3-DOX are toxic for Huh7 cells during prolonged treatments. AlamarBlue cell viability assay with Huh7 cells treated with 400 µg/ml GO and 100 µg/ml GO-Rg3-DOX, at different time points. All values were normalized to those obtained with untreated cells (grown in medium only). 10% DMSO was used as a positive control.

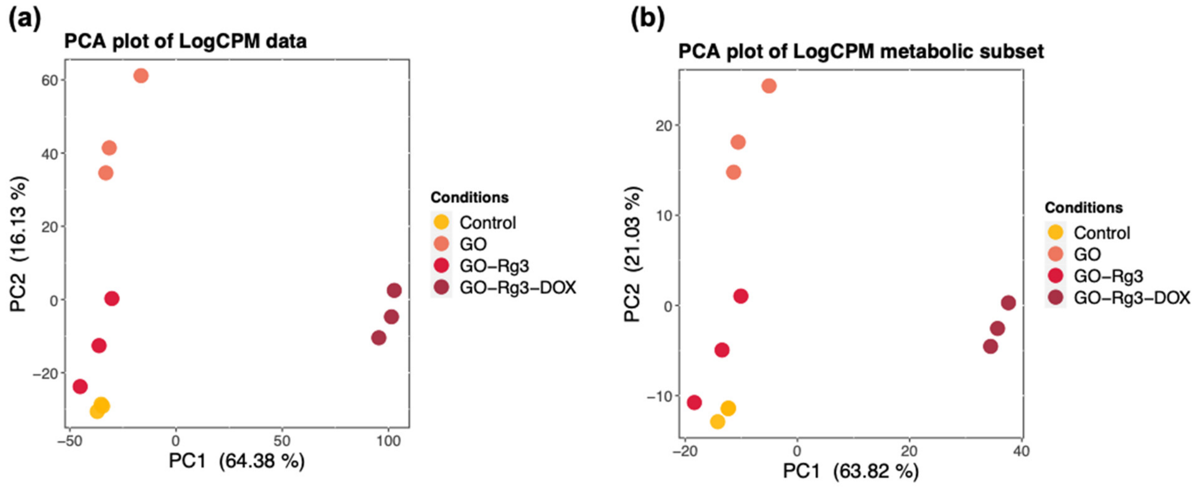

Figure S3. PCA plot of gene expression of Huh7 cells treated with GO, GO-Rg3, and GO-Rg3-DOX compared to untreated cells as the control. (**a**) Log-CPM of all genes were used in PCA analysis, (**b**) Log-CPM of only associated genes with metabolism were used. Points are colored for different treatments.

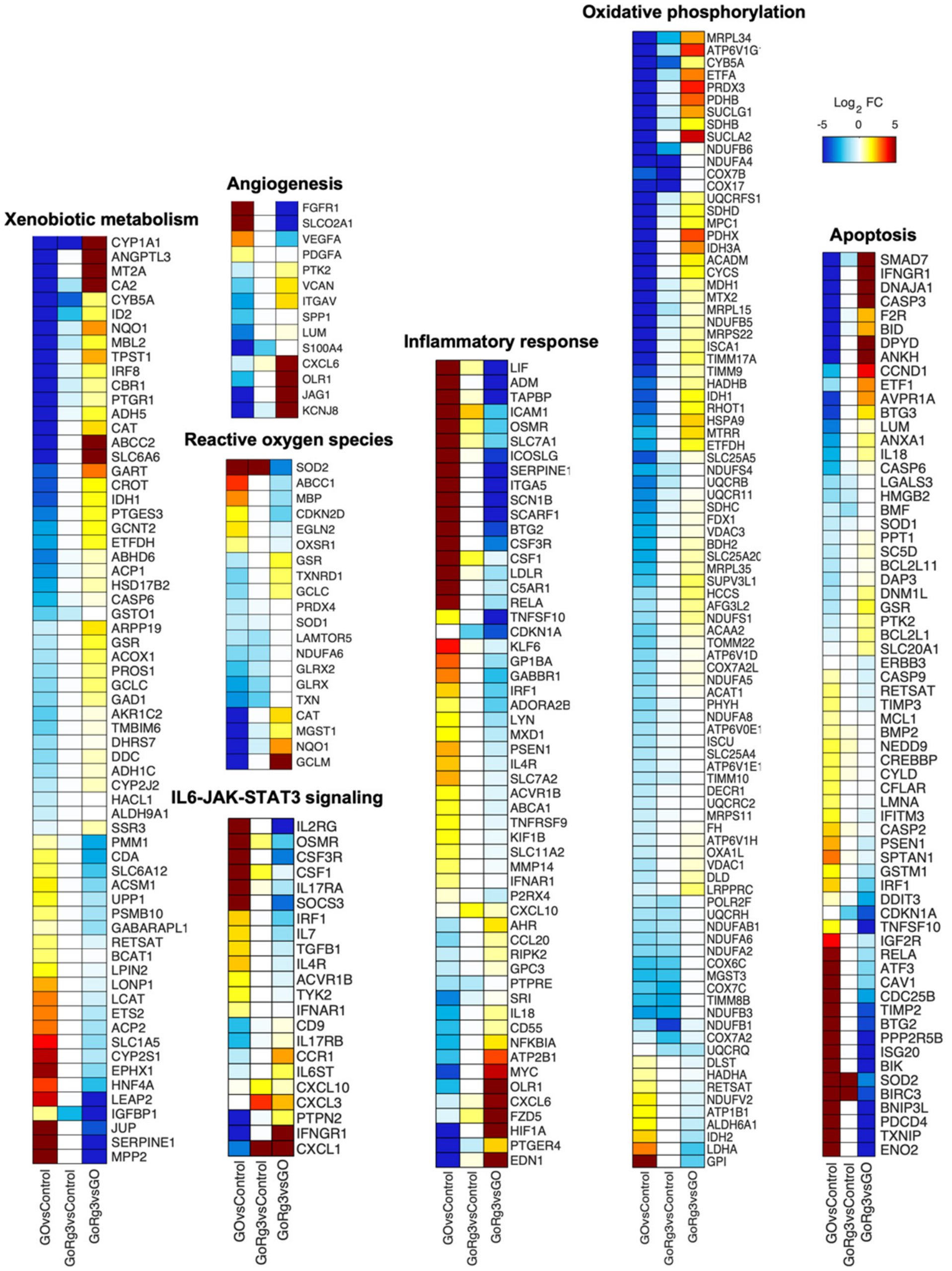

Figure S4. RNA sequencing supports mitigated toxicity of GO by Rg3 conjugation. Significantly changed genes in gene sets associated with the xenobiotic metabolism, reactive oxygen species, , IL6-JAK-STAT3 signaling, oxidative phosphorylation, inflammatory response, and apoptosis, are shown in the heatmaps, colored by log_2FC of the genes in GO-treated and GO-Rg3-treated compared with untreated samples, and also GO-Rg3-treated compared with GO-treated samples.

Log fold-change directionality (increase or decrease) information was incorporated with $\log_{10}$ (p_{adj}) for representing the significance of differential expressed genes in each gene set.

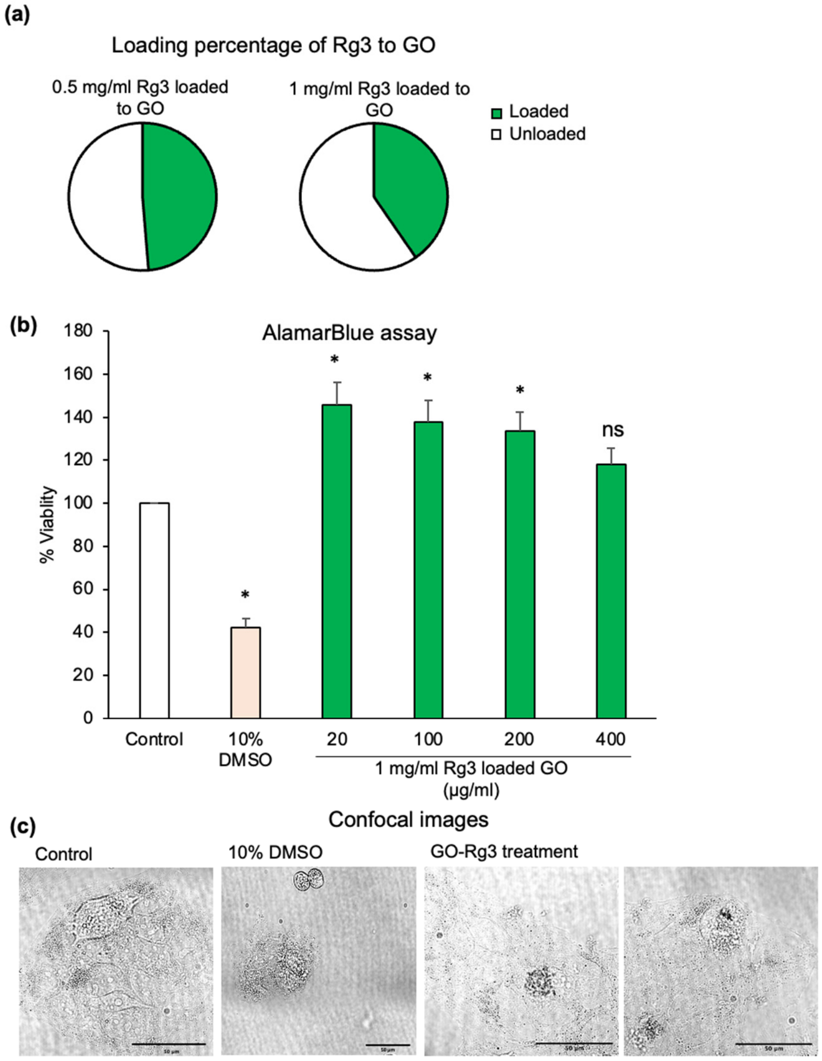

Figure S5. (**a**) Loading capacity of GO for Rg3. GO was conjugated with Rg3 at concentrations 0.5 and 1 mg/ml. The remaining material after GO loading with Rg3 was used for HPLC analysis. (**b**) AlamarBlue cell viability assay of Huh7 cells treated with GO-Rg3 for 24 h. GO-Rg3 was prepared using the 1 mg/ml solution of Rg3 for loading. Different concentrations of GO-Rg3 were administered. All values were normalized to those obtained from untreated cells (medium only). 10% DMSO was used as a positive control. Data represent the mean ±SE of three independent replicates and it was statistically analyzed and compared with the control (*: $P<0.05$, ns: not significant) using Student's t test. (**c**) Confocal imaging of Huh7 cells after treatment with our

standard preparation of GO-Rg3. Cell were incubated for 24 h in a medium containing 20 μg/ml GO-Rg3 and were subsequently washed with PBS before imaging. 10% DMSO was used as a positive control.

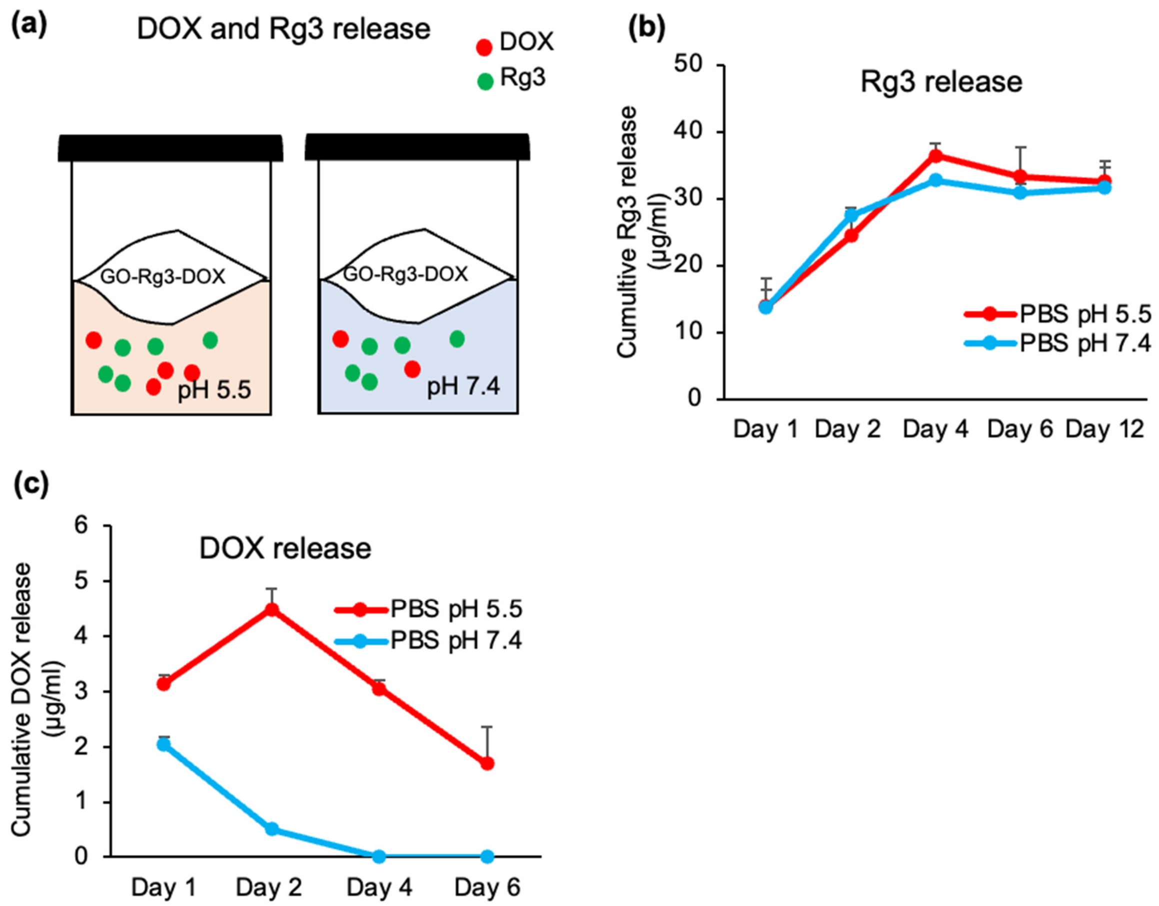

Figure S6. pH-dependent DOX and Rg3 release from GO-Rg3-DOX nanocarriers. (**a**) Schematic outline of the experimental set-up. (**b**) Cumulative Rg3 release over twelve days at pH 5.5 and pH 7.4. (**c**) Cumulative DOX release over six days at pH 5.5 and pH 7.4. The Rg3 and DOX release to PBS buffer was measured by HPLC.

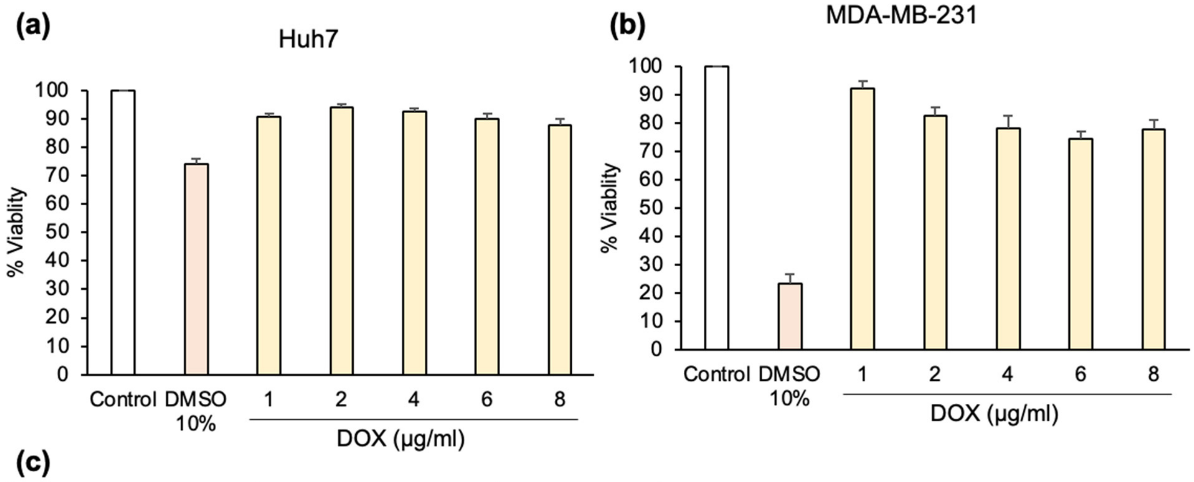

(c)

	IC50	References
Free DOX	5.8 µg/ml (10 µM)	[73]
Free DOX	9.9 µg/ml (17 µM)	[74]
Free DOX	4.7 µg/ml (8 µM)	[75]
GO-Rg3-DOX	<2 µg/ml (3 µM)	Our study

Figure S7. AlamarBlue viability assay of (**a**) Huh7 and (**b**) MDA-MB-231 cells treated with different concentrations of DOX for 24 h. All values were normalized to those obtained from untreated cells (medium only). 10% DMSO was used as a positive control. Data represent the mean ±SE of three independent replicates. (**c**) IC50 of DOX against Huh7 cells after 24 h treatment from other studies (black) and our study (red).

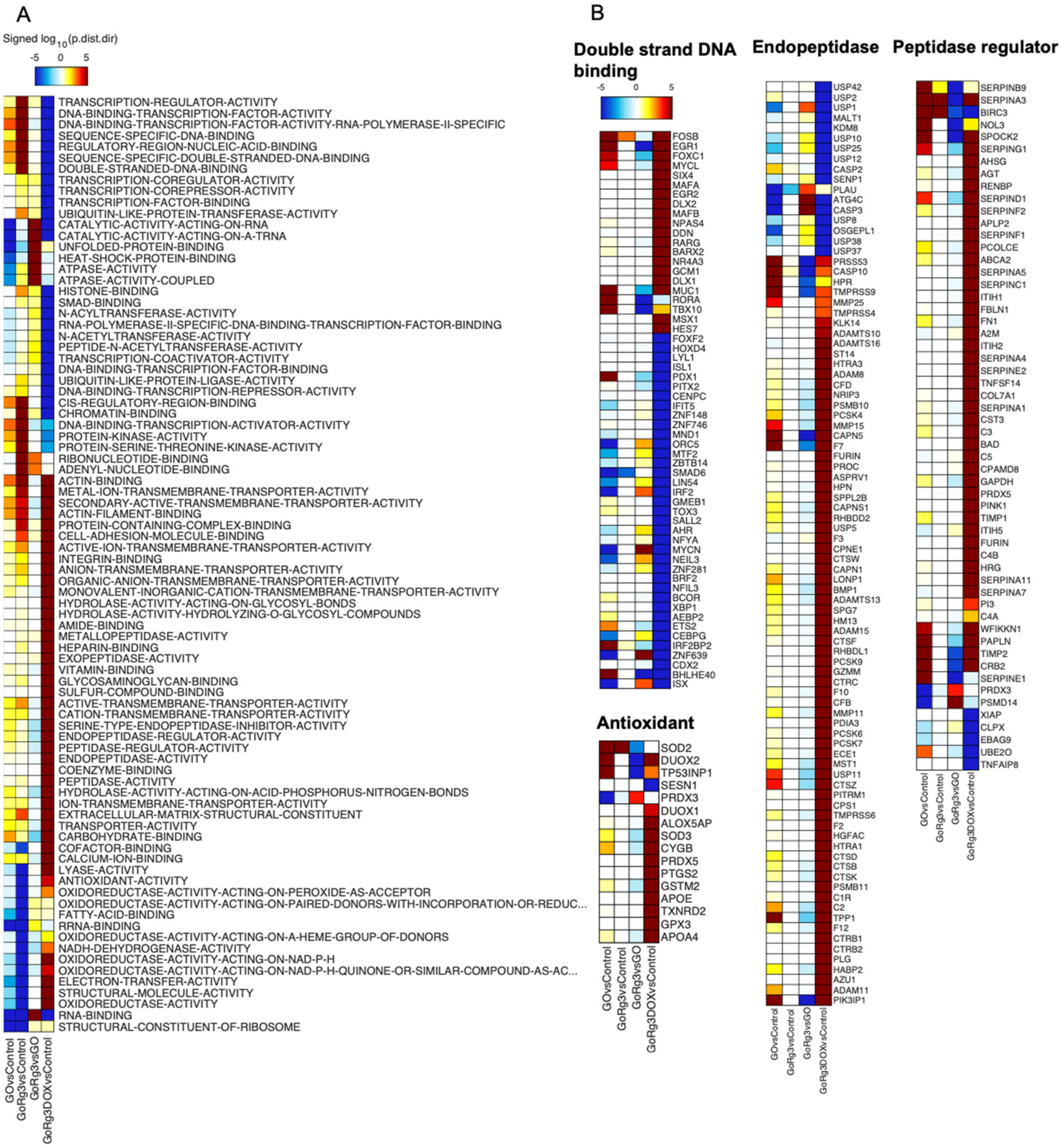

Figure S8. Effect of DOX delivered by GO-Rg3-DOX. A) Results of the directional GSA of DE analysis for GO, GO-Rg3, and GO-Rg3-DOX treated versus control samples. Only the molecular function (GO_terms) gene set collection retrieved from downloaded MSigBD database is shown here, and sets with <10 genes were excluded. The more significant (lower value) of the two directional P values for each gene set is shown in the heatmap as a $\log_{10}$-transformed value. The

distinct directional gene set P values ($p_{adj.dist.dir}$) are calculated for coordinated increases ($p_{adj,dist-dir-up}$) and decreases ($p_{adj,dist-dir-down}$) in expression. The value is also "signed," meaning that gene sets with a more significant decrease than increase ($p_{adj,dist-dir-down} < p_{adj,dist-dir-up}$) are negative; otherwise, they are positive. Only gene sets with a $p_{adj,dist-dir}$ less than 1e-6 in at least one of the comparisons are shown. B) Significantly changed genes in gene sets associated with double strand DNA binding, ion transmembrane transporter activity, endopeptidase activity, antioxidant activity, and peptidase regulator activity are shown in the heatmap, colored by log_2FC of the genes in GO, GO-Rg3, and GO-Rg3-DOX treated samples compared with untreated samples, and also GO-Rg3 compared with GO treated samples. The cut-off for all gene sets (except ion transmembrane transporter activity |log2FC| is > 2) |log2FC| is > 1. Log fold-change directionality (increase or decrease) information was incorporated with log_{10} (p_{adj}) for representing the significance of differential expressed genes in each gene set. Genes associated with these gene sets were extracted from molecular function gene set.

Table S1. Selected differentially expressed genes affected by GO-Rg3-DOX treatment compared with non-treated samples in gene sets associated with double strand DNA binding, endopeptidase activity, antioxidant activity, and peptidase regulator activity.

**Gene classification**	**Gene**	**Function**	**Expression**
Transcription regulatory	Intestine specific homeobox (*ISX*)	Function in development and differentiation	Under-expressed
Transcription regulatory	Basic helix-loop-helix family member e40 (*BHLHE40*)	Function in development and differentiation	Under-expressed
Transcription regulatory	Caudal type homeobox 2 (*CDX2*)	Function in development and differentiation	Under-expressed
Transcription regulatory	Interferon regulatory factor 2 binding protein 2 (*IRF2BP2*)	Function in development and differentiation	Under-expressed
Transcription regulatory	ETS proto-oncogene 2 (*ETS2*)	Function in development and differentiation	Under-expressed
Transcription regulatory	X-box binding protein 1 (*XBP1*)	Function in development and differentiation	Under-expressed
Transcription regulatory	Nuclear factor interleukin 3 regulated (*NFIL3*)	Function in development and differentiation	Under-expressed
Transcription regulatory	Zinc finger protein 281 (*ZNF281*)	Function in development and differentiation	Under-expressed
Transcription regulatory	BRF2 RNA polymerase III transcription initiation factor subunit (BRF2)	It is the general activator of RNA polymerase III transcription.	Under-expressed
Antioxidant activity genes	Glutathione peroxidase 3 (*GPX3*)	Antioxidant activity	Over-expressed
Antioxidant activity genes	Thioredoxin reductase 2 (*TXNRD2*)	Antioxidant activity	Over-expressed
Antioxidant activity genes	Dual oxidase 1 (*DUOX1*)	Antioxidant activity	Over-expressed
Antioxidant activity genes	Superoxide dismutase 3 (*SOD3*)	Antioxidant activity	Over-expressed
Antioxidant activity genes	Peroxiredoxin 5 (*PRDX5*)	Antioxidant activity	Over-expressed
Antioxidant activity genes	Glutathione S-transferase mu 2 (*GSTM2*)	Antioxidant activity	Over-expressed

Proteases with the apoptotic function	Granzyme M (*GZMM*)	A serine protease, that is constitutively highly expressed in natural killer (NK) cells and play a critical role in NK cell-mediated cytolysis [1]. In fact, increased expression of *GZMM* in GO-RG3-DOX samples may directly degrade inhibitor of caspase-activated DNase thereby release the nuclease activity of caspase-activated DNase for damaging DNA followed by apoptosis.	Over-expressed
Proteases with the apoptotic function	Hyaluronan binding protein 2 (*HABP2*)	A serine protease, functions as the tumor suppressor negatively regulating cell proliferation and cell migration [2]. Increased expression of *HABP2* might lead suppressing cell proliferation.	Over-expressed
Proteases with the apoptotic function	Caspase-10 (*CASP10*)	A thiol protease, that mediates extrinsic pathway of apoptosis by binding to the death-induced silencing complex (DISC) [3].	Over-expressed
Peptidase regulatory with apoptotic function	BCL2 associated agonist of cell death (*BAD*)	BAD promotes cell death.	Over-expressed
Peptidase regulatory with apoptotic function	TNF superfamily member 14 (*TNFSF14*)	It stimulates the proliferation of T cells, and trigger apoptosis of tumor cells.	Over-expressed
Peptidase regulatory with apoptotic function	TNF alpha induced protein 8 (*TNFAIP8*)	It acts as the negative mediator of apoptosis.	Under-expressed
Immune response	Azurocidin 1 (*AZU1*)	A glycoprotein packed with other proteins in azurophil granules of neutrophil that is an important multifunctional inflammatory mediator.	Over-expressed
Immune response	Signal peptide peptidase like 2B (*SPPL2B*)	A protease that localizes to endosomes, lysosomes, and the plasma membrane and cleaves the transmembrane domain of tumor necrosis factor alpha to release the intracellular domain, which triggers	Over-expressed

		cytokine expression in the innate and adaptive immunity pathways.	
Immune response	Cathepsin W (*CTSW*)	A member of the peptidase C1 family, is a cysteine proteinase that may have a specific function in the mechanism or regulation of T-cell cytolytic activity.	Over-expressed

Table S2. Coverage and quality of RNA sequencing (RNA-seq) results of samples used in this study. GRCh37 was used as the reference genome.

Sample ID	Total number of sequenced reads (M)	Total number of uniquely mapped reads (M)	RNA integrity number (RIN)	Ratio of all reads aligned to rRNA regions to total uniquely mapped reads (rRNA rate)	Ratio of exon-mapped reads to total uniquely mapped reads (Expression Profile Efficiency)	Total number of detected transcripts with reads ≥1
Control	42.9	39.4	9,60	0	88.5%	24,361
Control	34.9	32.4	8,90	0	86.6%	23,958
Control	48.5	44.3	9,50	0	88%	25,115
400 µg/ml GO	35.6	33.4	8,80	0	85.9%	23,737
400 µg/ml GO	43.3	40.1	9,70	0	86.5%	24,674
400 µg/ml GO	43.0	38.9	9,80	0	85.5%	24,737
400 µg/ml GO-Rg3	42.1	38.2	9,60	0	88.2%	24,063
400 µg/ml GO-Rg3	34.0	32.0	9,60	0	84.3%	24,196
400 µg/ml GO-Rg3	43.0	39.8	9,70	0	86.7%	24,873
100 µg/ml GO-Rg3-DOX	40.9	37.9	9,60	0	88.5%	24,940
100 µg/ml GO-Rg3-DOX	41.9	37.2	9,70	0	87.8%	26,524
100 µg/ml GO-Rg3-DOX	38.7	35.1	9,80	0	87.4%	25,403

[1] H. Lu, Q. Hou, T. Zhao, H. Zhang, Q. Zhang, L. Wu, Z. Fan, Granzyme M Directly Cleaves Inhibitor of Caspase-Activated DNase (CAD) to Unleash CAD Leading to DNA Fragmentation, The Journal of Immunology. 177 (2006) 1171–1178. https://doi.org/10.4049/jimmunol.177.2.1171.

[2] S.K. Gara, L. Jia, M.J. Merino, S.K. Agarwal, L. Zhang, M. Cam, D. Patel, E. Kebebew, Germline HABP2 Mutation Causing Familial Nonmedullary Thyroid Cancer, New England Journal of Medicine. 373 (2015) 448–455. https://doi.org/10.1056/NEJMoa1502449.

[3] F.C. Kischkel, D.A. Lawrence, A. Tinel, H. LeBlanc, A. Virmani, P. Schow, A. Gazdar, J. Blenis, D. Arnott, A. Ashkenazi, Death Receptor Recruitment of Endogenous Caspase-10 and Apoptosis Initiation in the Absence of Caspase-8, Journal of Biological Chemistry. 276 (2001) 46639–46646. https://doi.org/10.1074/jbc.M105102200.
